# Predicting survival and neurological outcome in out-of-hospital cardiac arrest using machine learning: the SCARS model

**DOI:** 10.1016/j.ebiom.2023.104464

**Published:** 2023-02-09

**Authors:** Fredrik Hessulf, Deepak L. Bhatt, Johan Engdahl, Peter Lundgren, Elmir Omerovic, Aidin Rawshani, Edvin Helleryd, Christian Dworeck, Hans Friberg, Björn Redfors, Niklas Nielsen, Anna Myredal, Attila Frigyesi, Johan Herlitz, Araz Rawshani

**Affiliations:** a Department of Molecular and Clinical Medicine, Institute of Medicine, Sahlgrenska Academy, University of Gothenburg, Gothenburg, Sweden; b Department of Anesthesiology and Intensive Care Medicine, Sahlgrenska University Hospital, Mölndal, Sweden; c Mount Sinai Heart, Icahn School of Medicine at Mount Sinai Health System, New York, NY, USA; d Karolinska Institutet, Department of Medicine, Karolinska University Hospital Danderyd, Stockholm, Sweden; e Prehospen-Centre for Prehospital Research, University of Borås, Borås, Sweden; f Department of Cardiology, Sahlgrenska University Hospital, Gothenburg, Sweden; g Wallenberg Laboratory for Cardiovascular and Metabolic Research, Institute of Medicine, University of Gothenburg, Gothenburg, Sweden; h The Lundberg Laboratory for Diabetes Research, Department of Molecular and Clinical Medicine, The Sahlgrenska Academy at the University of Gothenburg, 413 45, Gothenburg, Sweden; i Department of Clinical Sciences, Anesthesia & Intensive Care, Lund University, Skåne University Hospital, Malmö, Sweden; j Department of Clinical Sciences, Anaesthesia and Intensive Care, Helsingborg Hospital, Lund University, Lund, Sweden; k Department of Clinical Medicine, Anaesthesiology and Intensive Care, Lund University, Lund, SE-22185, Sweden; l The Wallenberg Centre for Molecular and Translational Medicine, University of Gothenburg, Gothenburg, Sweden

**Keywords:** Out-of-hospital cardiac arrest, Machine learning, Prediction model, Web application

## Abstract

**Background:**

A prediction model that estimates survival and neurological outcome in out-of-hospital cardiac arrest patients has the potential to improve clinical management in emergency rooms.

**Methods:**

We used the Swedish Registry for Cardiopulmonary Resuscitation to study all out-of-hospital cardiac arrest (OHCA) cases in Sweden from 2010 to 2020. We had 393 candidate predictors describing the circumstances at cardiac arrest, critical time intervals, patient demographics, initial presentation, spatiotemporal data, socioeconomic status, medications, and comorbidities before arrest. To develop, evaluate and test an array of prediction models, we created stratified (on the outcome measure) random samples of our study population. We created a training set (60% of data), evaluation set (20% of data), and test set (20% of data).

We assessed the 30-day survival and cerebral performance category (CPC) score at discharge using several machine learning frameworks with hyperparameter tuning. Parsimonious models with the top 1 to 20 strongest predictors were tested. We calibrated the decision threshold to assess the cut-off yielding 95% sensitivity for survival. The final model was deployed as a web application.

**Findings:**

We included 55,615 cases of OHCA. Initial presentation, prehospital interventions, and critical time intervals variables were the most important. At a sensitivity of 95%, specificity was 89%, positive predictive value 52%, and negative predictive value 99% in test data to predict 30-day survival. The area under the receiver characteristic curve was 0.97 in test data using all 393 predictors or only the ten most important predictors. The final model showed excellent calibration. The web application allowed for near-instantaneous survival calculations.

**Interpretation:**

Thirty-day survival and neurological outcome in OHCA can rapidly and reliably be estimated during ongoing cardiopulmonary resuscitation in the emergency room using a machine learning model incorporating widely available variables.

**Funding:**

10.13039/501100004359Swedish Research Council (2019–02019); Swedish state under the agreement between the Swedish government, and the county councils (ALFGBG-971482); The 10.13039/501100017018Wallenberg Centre for Molecular and Translational Medicine.

**Research in context**

**Evidence before this study**

While up to 30% of all cases of out-of-hospital cardiac arrest (OHCA) achieve return of spontaneous circulation (ROSC), only 10% survive, such that a substantial proportion undergo difficult and potentially long intensive care with death as the outcome. OHCA is a leading cause of death and the cost of OHCA is staggering at all levels. Predicting survival and neurological function in OHCA is very difficult for physicians, and there are few tools to aid the decision process.

**Added value of this study**

Using over 55,615 cases of OHCA, for whom we had almost 400 predictors of survival, we have developed SCARS-1, an operational machine learning model that enables clinicians to calculate survival and neurological function in <15 s with an area under the receiver operating curve (AUROC) of 0.95, with excellent calibration. Additionally, we developed the SCARS-1 web application (freely available for download) that enables intuitive and easy access to the machine learning model.

**Implications of all the available evidence**

OHCA carries a poor prognosis and a majority of OHCA patients transported to the emergency department without ROSC do not survive. The SCARS-1 prediction model and web application can assist the clinician in the ED by offering a robust prediction of survival and neurological function within <15 s.

## Introduction

Out-of-hospital cardiac arrest (OHCA) is common and associated with poor survival rates of 5–10%. According to the EuReCa TWO study,^1^ Cardiopulmonary resuscitation (CPR) is started in approximately 68% of OHCA cases confirmed by the emergency medical service (EMS). Among those who received CPR, resuscitation efforts were terminated prehospitally in 64% of the cases, while the remaining 36% were transported to a hospital, and among those admitted, roughly one in four survived. Overall survival in OHCA was 8%. Rephrased, most OHCA cases transported to hospital die despite prehospital and in-hospital resuscitation efforts.

While chest compressions are effective even in the hands of laymen,^2^ the decision to terminate CPR should lie in the hands of experienced physicians and EMS personnel, after careful consideration of the likelihood of survival and neurological prognosis. Regrettably, humans are at best poor at probabilistic calculations.^3^^,^^4^ Given the discrepancy between rates of return of spontaneous circulation (ROSC) and 30-days survival – which may differ up to 10-fold – it is desirable to develop a clinical prediction model for prediction in the emergency department (ED).^5^

We used the Swedish Registry for Cardiopulmonary Resuscitation (SRCR) to develop a machine learning derived clinical prediction model using 393 candidate predictors in 55,615 cases of OHCA. Feature selection and model specification was entirely data-driven. The final model was deployed as a web application and required less than 30 s to obtain real-time predictions using the machine learning model. The model was developed using variables available no later than patient arrival in the ED, which marks the potential point of use.

## Methods

### Study population

We used the SRCR to include all cases of OHCA from 2010 to 2020. The registry has been described previously.^2^^,^^6^ For a description of the Swedish EMS system as well as the in-hospital organization, refer to supplementary discussion 1. The SRCR is a nationwide quality registry and was launched in 1990. Since 2008, all ambulance organizations in Sweden reported OHCA cases to the registry. Data reporting to the SRCR follows the Utstein style. We included all OHCA patients aged 18 years and older, where resuscitation was attempted from Jan 1st, 2010 to Dec 31st, 2020.

An OHCA is defined as a cardiac arrest occurring in non-hospitalized patients. The first recorded rhythm is defined as either shockable (ventricular fibrillation or pulseless ventricular tachycardia (VF/pVT) or non-shockable pulseless electrical activity (PEA) or asystole). Time delays from collapse to the emergency call, start of CPR, delivery of defibrillations, dispatch of EMS, and the arrival of EMS at the scene are reported. Additional variable details are provided below. Information regarding DNAR orders is not recorded in the SRCR and therefore not included in the study.

### Data merger

We merged the SRCR with the Swedish Inpatient and Outpatient Registry. The Inpatient Registry contains all inpatient records since 1987 and has been validated.^7^ The Outpatient Registry contains all outpatient clinic visits since 2002. The primary and up to 20 secondary diagnoses are available in each registry. Diagnoses are classified using the International Classification of Disease (ICD) versions 9 and 10, with data retrieved from 1987 for the Inpatient Registry and 2002 in the Outpatient Registry. The categorization in the SRCR of causes of OHCA has changed over time, both categorizations are reported.

Medications were retrieved from the Swedish Prescribed Drug Registry, including all prescriptions registered and expedited since 2005. In addition, we retrieved prescriptions registered from Jan 1st, 2008, according to the Anatomical Therapeutic Chemical (ATC) classes.

The LISA (integrated longitudinal database for health insurance and labor market studies) database was used to obtain socioeconomic data, e.g., income, education, country of birth, housing conditions, etc. We only used data recorded during the year before cardiac arrest to avoid reverse causation between socioeconomic status and cardiac arrest (e.g., income may be dramatically reduced after cardiac arrest).

### Candidate predictors

After merging the above-mentioned data sources, we had 393 predictors. These included seven predictors describing the circumstances of the cardiac arrest, six critical time intervals during the cardiac arrest, four predictors describing patient demographics, six predictors describing initial presentation at EMS arrival two geographical predictors, nine predictors on spatiotemporal data during arrest, 3 predictors on socioeconomic status, 18 medication classes, 328 comorbidities from the Swedish in- and outpatient registry. For a description of included variables relating to comorbidities and medications, see Table S1. For a flow chart of included variables, refer to Fig. S1.

### Outcome measures

The primary outcome measure was survival at 30 days. The secondary outcome measure was neurological function measured using cerebral performance category (CPC) score at discharge (1, no or mild sequelae; 2, moderate sequelae; 3, severe sequelae; 4, vegetative state; 5, brain dead).

### Role of the funding source

The funder of the study had no role in study design, data collection, data analysis, data interpretation, or writing of the report.

### Ethics

The study complies with the declaration of Helsinki and was approved by the Swedish Ethical Review Authority (No 2020-02017). The need for informed consent was waived by the Ethical Review Authority due to the retrospective nature of the study.

### Statistics

#### Descriptive statistics

Patient characteristics are described using frequencies and relative frequencies. No inferences are made from baseline data, representing the entire population with OHCA in Sweden during the period. We only present the 15 most common coexisting conditions and medications.

### Machine learning

#### Model training and testing

To develop, evaluate and test an array of prediction models, we created stratified (on the outcome measure) random samples of our study population. We created a training set (60% of data), evaluation set (20% of data), and test set (20% of data). We used the whole study population to split into train, evaluation and test sets. For a description of software refer to supplemental discussion 2.

#### Model evaluation and comparison

We used 5-fold cross-validation, repeated five times to compare the competing models. Comparisons across models were made using the area under the receiver operating characteristic (ROC) curve.

#### Addressing imbalance

For binary classification problems, the relative frequencies of the classes can have a significant impact on model performance.^8^ An imbalance is evident in the SRCR since approximately 10% of all patients survive. We addressed imbalance by down-sampling the number of deceased individuals, such that for one survivor, we included four deceased individuals, which reduced class imbalance. Only the training data set was artificially balanced. The evaluation and test sets were unaltered to assess model performance on representative data.

#### Candidate model frameworks

We initially considered logistic regression, support vector machines, neural networks, gradient boosting, extreme gradient boosting, and random forest as candidate prediction models. Frameworks requiring hyperparameter tuning (gradient boosting, extreme gradient boosting, random forest, SVM, neural networks) were tuned using manual grid. Comparisons were made using the area under the ROC curve (AUC).

Extreme gradient boosting (XGB) outperformed all models. We tuned the XGB model using a grid of 144 different hyperparameter combinations (Fig. S2). Still, we noted additional improvement in AUC was possible and computed 14 additional models, which showed no further improvement. During hyperparameter tuning, we tuned eta (step size shrinkage), gamma (minimum loss reduction required to further partition) on a leaf node, rounds (number of trees grown), max depth (tree depth), column sample by tree (proportion of columns sampled for each tree), min child weight (minimum sum of instance weight needed in a child) and subsample (proportion of patients selected for each tree) (Table S2 Grid search for hyperparameter tuning).

#### Tuning of decision threshold

We derived new cut-offs for the classification (decision) threshold using the evaluation set. The conventional 50% probability threshold may be suboptimal for the current prediction task. An optimal prediction model would have 100% sensitivity and 100% specificity for predicting survival. Low sensitivity could theoretically result in patient harm since the model may predict death in a patient who could survive. We, therefore, tuned our threshold to maximize sensitivity for survival. The threshold was calibrated using the ROC curve. This was done by maximizing the Youden index, which measures the proportion of correctly predicted samples for both the survival and deceased groups. The Youden index can be computed for each cut-off on the ROC curve, the cut-off that maximizes the Youden index represents the optimal model. In addition, we also identified the cut-off that yielded a sensitivity of 95%.

#### Relative predictor importance

Because the model included 393 predictors, ranging from coexisting conditions to prehospital interventions performed by the EMS, we calculated the relative importance of all predictors. This clarifies which predictors are the most important for determining survival in OHCA. The top 20 predictors were then used to create and evaluate parsimonious models (XGB) with 1–20, in order of importance, predictors. The purpose of this was to determine how many predictors were required to achieve an acceptable precision to deploy a clinically feasible web application.

#### Calibration

Model calibration was evaluated by comparing observed probabilities versus predicted probabilities.

#### Missingness

We imputed missing values using a non-parametric method combining random forest with predictive mean matching.^9^^,^^10^ For a description of the degree of missingness for the to 20 predictors, see Table S3.

#### Web application

The final model was deployed as a web application (Fig. S5).

### Role of the funding source

The funder of the study had no role in study design, data collection, analysis, interpretation, writing of the paper or decision to submit for publication.

## Results

A total of 55,615 cases of OHCA were recorded in the SRCR from 2010 to 2020. Table 1 shows the baseline characteristics of the study population (Table S4 shows the same cohort stratified by survival at 30 days). The mean age was 69 years, and 34% were women. Heart disease caused 63% of all cases. Cardiac arrest occurred at home in 72% of all cases, 65% were witnessed, and 55% received bystander CPR. The initial rhythm was pVT/VF in 23% of cases, and 34% achieved ROSC. Table 2 shows the outcome measures of the entire cohort. The 30-day survival was 12%, 91% with CPC 1-2. The one-year survival was 10%. Fig. S6 shows crude rates of ROSC and hospitalization.

**Table 1:** Baseline characteristics of 55,615 patients with out of hospital cardiac arrest.

Characteristic	
Patients, n	55,615
Age yr (mean) (SD)	68.88 (17.92)
Female Sex, n (%)	18,881 (34.0)
**Cause of OHCA old categorizations – n (%)**	
Cardiac	30,816 (62.5)
Overdose	1427 (2.9)
Accident	1139 (2.3)
Pulmonary disease	2765 (5.6)
Asphyxi	1286 (2.6)
Suicide^a^	1121 (2.3)
Drowning	465 (0.9)
Sudden infant death	170 (0.3)
Other	10,155 (20.6)
**Cause of OHCA, new categorizations – n (%)**	
Medical condition	45,703 (90.5)
Trauma	1249 (2.5)
Intoxication	1520 (3.0)
Drowning	489 (1.0)
Electrical accident	2 (0.0)
Asphyxia	1546 (3.1)
**Time of OHCA – n (%)**	
0–6 am	7630 (16.5)
1–6 pm	14,015 (30.3)
7–11 pm	8847 (19.1)
7–12 am	15,809 (34.1)
**Location of cardiac arrest – n (%)**	
Home	39,661 (71.6)
Public location	8989 (16.2)
Other locations	6728 (12.1)
OHCA during sports – n (%)	1724 (3.1)
**Critical time intervals, minutes – median (IQR)**	
Collapse to EMS dispatch	2.00 [1.00, 5.00]
Collapse to CPR	3.00 [0.00, 10.00]
Collapse to first defibrillation	15.00 [8.00, 24.00]
Time from collapse to EMS arrival	13.00 [8.00, 20.00]
Time from EMS dispatch to EMS arrival	10.00 [7.00, 16.00]
Time to ROSC	15.00 [9.00, 23.00]
**Circumstance at time of CA – n (%)**	
Witnessed CA	34,923 (64.7)
CA witnessed by EMS	3916 (21.3)
Telephone CPR	9565 (62.7)
Training CPR provider	
Layperson without CPR training	5389 (46.9)
Layperson with CPR training	4215 (36.7)
Health care worker	1891 (16.5)
EMS first on scene	22,255 (74.5)
Fire fighters first on scene	8940 (31.1)
Police first on scene	1373 (4.9)
**Initial presentation et EMS arrival – n (%)**	
**Initial Rhythm**	
VF/pVT,	11,374 (23.2)
PEA,	8421 (17.2)
Asystole	29,248 (59.6)
Patient conscious at EMS arrival at scene	5728 (10.6)
Patient breathing at EMS arrival at scene	
No breathing	42,007 (77.7)
Agonal breathing	5791 (10.7)
Normal breathing,	6212 (11.5)
Unknown,	22 (0.0)
Pulse at EMS arrival at scene	7384 (14.0)
**Prehospital interventions – n (%)**	
Bystander treatment before EMS arrival - Yes	29,491 (55.1)
CPR before EMS arrival -Yes	13,488 (57.3)
Public AED attached to patient – Yes	1863 (6.6)
Bystander def. with public AED – Yes	652 (36.1)
Mechanical CPR	21,486 (40.4)
Ventilation	51,142 (92.6)
Endotracheal intubation	15,437 (28.3)
Laryngeal Mask	19,960 (61.0)
Defibrillation	17,812 (33.3)
Number of defibrillations, n (mean (SD))	3.48 (3.16)
Adrenalin	43,290 (78.8)
Amiodarone	6365 (11.8)
Return of spontaneaus circulation at hospital arrival	14,190 (44.7)
Conscious at hospital arrival	3418 (11.0)
**In-hospital interventions, n (%)**	
Coronary angiography	294 (37.4)
Percutaneaus coronary intervention	3285 (29.4)
Coronary artery bypas surgery	190 (1.7)
ICD implanted	1204 (10.9)
ECMO	21 (2.7)
**Coexisting conditions,** n (%)	
Hypertension,	24,821 (44.6)
Fall associated with the CA	19,323 (34.7)
Heart failure	12,669 (22.8)
Ischemic heart disease	11,505 (20.7)
Atrial flutter/fibrillation	11,363 (20.4)
Type 2 Diabetes Mellitus	10,609 (19.1)
Arthrosis	8710 (15.7)
Hyperlipidemia	8699 (15.6)
Trauma to the head associated with the CA	8506 (15.3)
Angina pectoris	8492 (15.3)
Epilepsy	8350 (15.0)
Malignancy	7941 (14.3)
**Medications used prior to OHCA, n (%)**	
Anticoagulants	20,126 (36.2)
Beta blockers	18,640 (33.5)
ACE-inhibitors	18,199 (32.7)
Diuretics	15,020 (27.0)
Hyperlipidemia medications	13,221 (23.8)
Antiacids	11,143 (20.0)
Calcium channel blockers	8838 (15.9)
Diabetes medications	8300 (14.9)
Antibiotics	7730 (13.9)
Antiarrhytmics	6616 (11.9)
Hormone modulators	1490 (2.7)
Other hypertension medications	632 (1.1)

EMS = emergency medical service (ambulance). VF/VT = ventricular fibrillation, ventricular tachycardia. ECMO = extracorporeal membrane oxygenation. ICD = Implantable Cardioverter Defibrillator.

a Cause of cardiac arrest was reported as an attempt to suicide reported in the Swedish registry for CPR.

**Table 2:** Outcomes in 55,615 patients with out-of-hospital cardiac arrest.

Outcome, n (%)	
Return of spontaneaus circulation (ROSC)	18,234 (34.4)
**Hospital admission**	
Not admitted to hospital	16,362 (29.6)
Admitted to hospital	11,781 (21.3)
Unknown	27,182 (49.1)
Discharged alive (of hospital admitted pts)	5359 (46.1)
Death before end of follow-up	51,178 (92.0)
Death within 30 days	49,424 (88.9)
Death at 1-year follow-up	50,070 (90.0)
**CPC score, n (%)**	
CPC 1	3433 (64.4)
CPC 2	686 (12.9)
CPC 3	295 (5.5)
CPC 4	109 (2.0)
CPC 5	14 (0.3)
Missing	794 (14.9)

CPC = cerebral performance category. EMS = emergency medical service (ambulance). VF/VT = ventricular fibrillation, ventricular tachycardia. ECMO = extracorporeal membrane oxygenation. ICD = Implantable Cardioverter Defibrillator.

### Data sets, features, and hyperparameter tuning

A total of 393 predictors were included in the models (using the same set of features for all models). All features not included in the baseline tables are described in Table S1.

The training set included 33,370 patients, the evaluation set 11,123 patients, and the test data included 11,122 patients. After down sampling, the number of deaths in the training data included 18,575 patients, of whom 3715 survived and 14,860 died. The grid for hyperparameter tuning is presented in Table S2.

### Model selection

The best model encompassed 1400 trees. The maximum tree depth was 10, shrinkage was 0.01, gamma (minimum loss reduction) was 0, column samples for each tree were 0.8, and the minimum sum of instance weight needed in a child 1, and subsample ratio was 0.7. The difference between various models was negligible. The performance of the final model in the evaluation set was AUC 0.97. With the standard decision threshold of 50% (for classification of dead vs. alive), the sensitivity was 0.82, specificity 0.97, PPV 0.80, and NPV 0.98.

### Relative importance

**Fig. 1:**
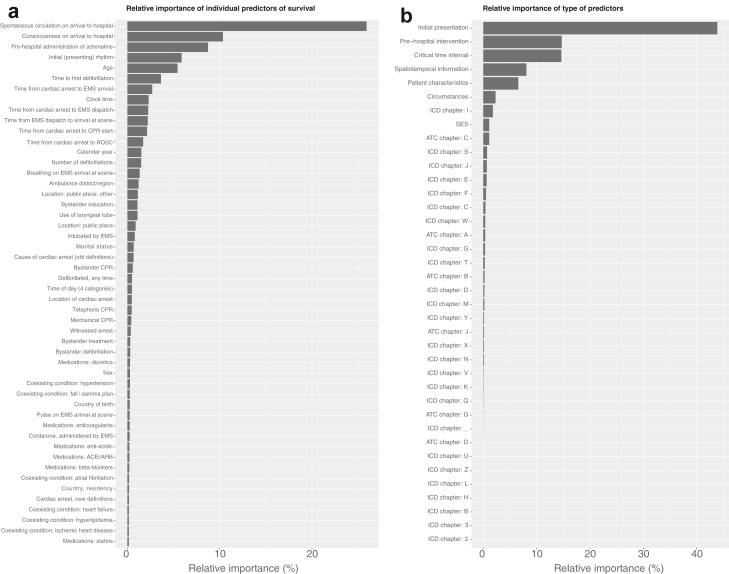
(a): Relative variable importance plot: Top 50 predictors ranked by relative importance to 30-day survival. N=55,615. (b): Relative variable importance plot: 39 predictor categories ranked by relative importance to 30-day survival. N=55615. SOS= socioeconomic status, ICD= International classification of diseases, ATC= Anatomical, Therapeutic, Chemical classification.

Fig. 1a shows the relative importance of the top 50 predictors (among all 393). Variables that were available in the SRCR dominated in terms of importance. Among variables not specific to the SRCR, only calendar year and age were among the top predictors. Variables concerning the initial presentation, age, use of adrenaline, and critical time intervals were the most important predictors. Coexisting conditions were much less important, as were medications, socioeconomic status, and circumstances around the cardiac arrest. Fig. 1b shows all predictors collapsed into categories of predictors. This figure shows that the emergency department (ED) presentation, prehospital interventions, and critical time intervals dominated the importance.

### Best predictors

**Fig. 2:**
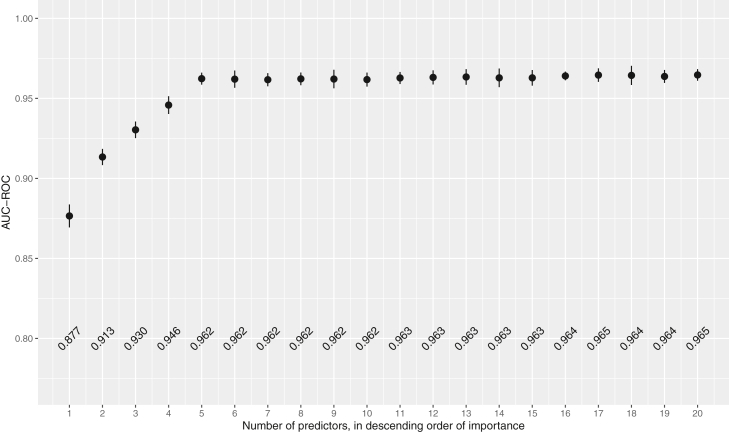
AUC-ROC with corresponding 95% confidence intervals for prediction of 30-day survival for 20 models including the top 1 to top 20 predictors of survival. AUC-ROC: Area Under the Curve - Receiver Operator Characteristics curve.

Refitting the final model with the top 1 to 20 predictors, in sequential order, shows that the AUC values increased rapidly when adding the first few top predictors. Still, little AUC was gained after including additional predictors (Fig. 2). There was no difference between the top 10 and 393 predictors regarding AUC (p = 0.20).

The top 20 predictors were related to the initial presentation, prehospital interventions, and critical time intervals (i.e., data available to the physicians in the ED). The model performance on training data and test data regarding CPC-score can be seen in Fig. S3.

### Tuning the decision threshold: evaluation data set

**Fig. 3:**
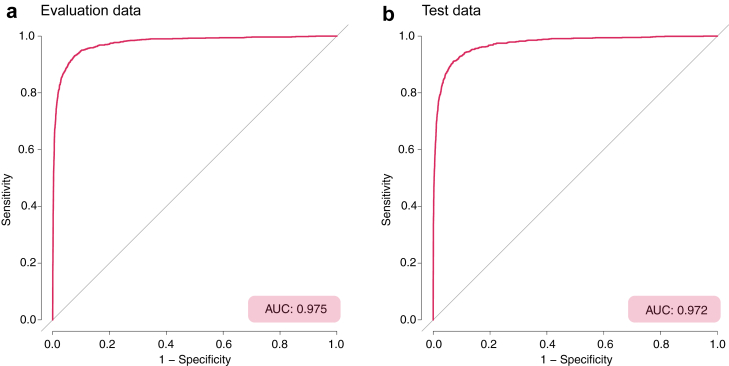
Receiver operating characteristic (ROC) curve for prediction of 30-day survival based in (a) evaluation data and (b) test data. AUC-ROC: Area Under the Curve - Receiver Operator Characteristics curve.

Using the standard decision threshold (50% cut-off) sensitivity was 77%, specificity 98%, positive predictive value (PPV) 86%, negative predictive value (NPV) 97%. The Youden index suggested a decision threshold at 7.9%, which yielded a sensitivity of 92%, specificity of 92%, a PPV of 60%, NPV of 99%. To achieve a sensitivity of 95%, the decision threshold was set at 4.6% probability of survival, resulting in sensitivity 95%, specificity 89%, PPV 52%, and NPV of 99%. Refer to Fig. 3 for AUC-ROC on evaluation data and Table S5 for details.

### Model performance on test data

The default cut-off (50%) yielded a sensitivity of 76% and specificity of 98%. The cut-off identified with the Youden index yielded a sensitivity of 92%, specificity 92%, PPV 58%, and NPV 98.9%. The 4.6% cut-off yielded a sensitivity of 94%, specificity 89%, PPV 52% and NPV 99%. See Fig. 3 for AUC-ROC on test data and Table S6 for model performance on test data.

### Calibration

**Fig. 4:**
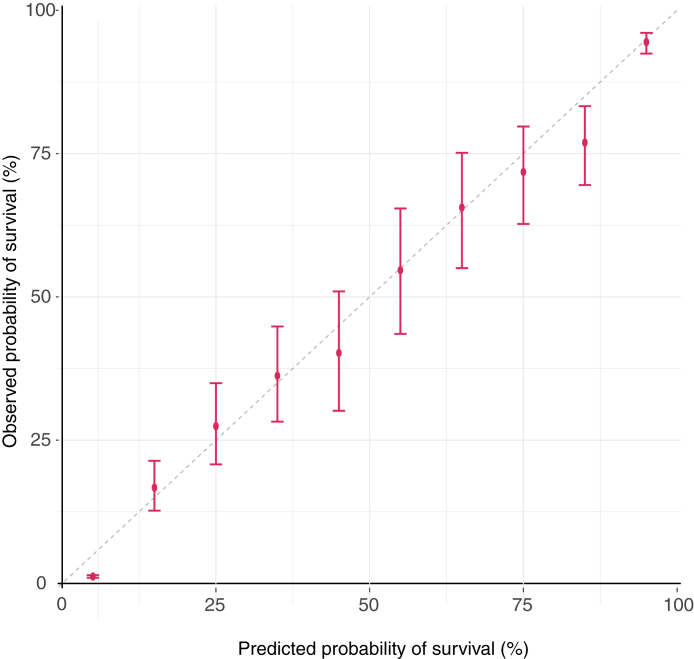
The calibration plot illustrates the agreement between predicted probabilities and observed probabilities of survival with 95% CI.

The calibration plot is presented in Fig. 4. The final model showed excellent calibration across the spectrum of survival probabilities.

### Web application

The web application is available at https://gocares.se. Refer to Fig. S4 for a step-by-step tutorial on the use of the application with 3 example patients.

## Discussion

Rapid and accurate probability calculations are critical in the setting an out-of-hospital cardiac arrest. The probability of survival, selection of interventions, likely underlying cause, etc., are essential information needed in seconds. Good prediction models can save lives and reduce suffering and costs by avoiding futile resuscitation efforts. Death due to cardiovascular causes is a public health issue associated with more deaths than any cancer.^11^ Out-of-hospital cardiac arrest is managed by ambulance and hospital staff with varying knowledge and experience. We have developed a clinical prediction model with good performance using a data-driven machine learning approach. It utilizes predictors that can be collected in most health care settings.

Our prediction model was derived using 393 candidate predictors in 55,615 cases of OHCA to predict 30-day survival. The top 20 predictors were related to the initial presentation, prehospital interventions, and critical time intervals, i.e., readily available to the physicians in the ED. This is a fundamental aspect of the model; limiting the variable inclusion at the time of arrival to the ED results in a prediction model that can be used immediately upon ambulance arrival to the hospital. Moreover, the model is dominated by relatively few predictors, making it possible to predict with very high precision using only 5 to 10 predictors.

The developed web application uses the final prediction model to calculate survival, and entering all variables for one patient takes approximately 30 s, which highlights the clinical potential for such models.

A few similar studies merit further discussion. Seki et al.^12^ developed a machine learning algorithm for one-year survival following OHCA using 53 predictors. The model showed good performance (AUC 0.94). Still, the usefulness of the prediction tool was limited since only OHCA of presumed cardiac etiology were included, and it is improbable that any clinician will be able to enter data on 53 variables during ongoing CPR. Kwon et al.^13^ used multiple machine learning methods in addition to logistic regression models to predict survival following OHCA among patients that achieved ROSC. Although the model showed high AUC (0.95) by only including patients with ROSC, the usefulness of such a score in the ED is limited since most patients with sustained ROSC are transferred to the ICU for further treatment, and prognostication is often made at a later stage. The same limitation applies to several other prediction tools (Miracle2,^14^ OHCA,^15^ TTM,^16^ CAHP^17^) – they can be used to predict neurological function among patients with OHCA and ROSC, but they do not help the clinician with a patient with OHCA and no ROSC in the ED. We present the only prediction model for OHCA with an AUC over 0.95 using only readily available variables.

The decision to continue or terminate resuscitation should be made based on all available information: assessing medical futility, defining an unfavorable outcome, and incorporating the patients' views on the quality of life (when known) and what to the patient considered a life worth living. Medical futility has been defined as having less than a one percent chance of survival.^18^ This definition has received criticism for not considering functional outcomes and being biased towards some socioeconomic, demographic, and cultural factors and groups. Relying on one prediction tool comes with the risk of self-fulfillment, and existing ethical guidelines recommend taking multiple factors into account.^5^ This model hereafter referred to as SCARS (Swedish Cardiac Arrest Risk Score), estimates the chance of survival and neurological function based on an entire nation's collected data and can be used as an adjunct to other available information. The resuscitation team can incorporate the estimated survival probability with less quantifiable data, such as frailty, the patient's personal views, quality of life, and previously stated opinions and wills, to make an informed decision to continue or terminate resuscitation. The SCARS model does not suggest whether or not CPR should be discontinued; it merely calculates the probability of survival. We recommend that the decision to withhold or withdraw resuscitation be context-specific and note that all tools intervening in patient management must be evaluated in clinical trials before clinical implementation.

Most cardiac arrests occur in the out-of-hospital setting, and only 5–10% of the patients in whom resuscitation is initiated survive.^1^ If the OHCAs where resuscitation was never initiated are included, the number of survivors is below 5%; i.e., in more than 95% of OHCA cases, the decision to withhold or withdraw resuscitation is made. There is a significant variation in prehospital Termination of Resuscitation (TOR) between EMS systems,^19^^,^^20^ ranging from 0% to over 50% prehospital TOR. This variability is likely due to different policies and practices regarding the termination of resuscitation and not due to medical reasons. The use of a validated and reliable prehospital and emergency department prediction tool could result in patients being treated more uniformly regardless of location.

Conflicting evidence exists as to whether experienced clinicians are better at predicting survivors from non-survivors than existing scoring systems (APACHE, SAPS, MPM).^21^ ICU physicians were better than existing scoring systems when assessing survival 24 h post ICU admission. Still, both the physicians and the scoring systems were only moderately accurate at outcome prediction.^22^ Data suggests that estimates of prognosis are often based on experience and clinical reasoning skills rather than objective information,^23^^,^^24^ which can sometimes be misleading. Data from physician estimates of prognosis in terminally ill patients showed that physician estimates of time to death were only correct 20% of the time (within 33% of actual survival), over-optimistic predictions (63%) were common and overall physicians overestimated survival time by a factor of 5.^23^^,^^25^ To get an approximation of how difficult estimation of survival probability can be, we asked 17 physicians working in the ICU and CCU (unpublished data) to estimate the chance of survival in four different OHCA scenarios. The clinicians’ estimates deviated markedly (up to a factor 3) from crude and predicted survival probabilities, suggesting that prediction tools are needed.

A strength of the present study is the extensive, representative data set, the many included parameters, and the large number of models evaluated. The SCARS prediction tool reliably predicts survival following OHCA, and the benefits of a well-functioning and reliable tool for patients, health care workers, and society are potentially of great importance. In addition, future research should prospectively and externally validate the prediction tool using data from other nationwide out-of-hospital cardiac arrest registries.

### Limitations

Our prediction model cannot be used in the prehospital setting since it includes two predictors recorded at hospital arrival (consciousness and circulation on hospital arrival). Our model should neither be used in patients with ROSC or consciousness since they should always be admitted to the appropriate level of care. However, including these predictors is critical since they were the strongest survival predictors, and they are readily available to the clinician in the ER. Our prediction model has not been validated in other countries and should be used with caution elsewhere. However, the SRCR includes over 95% of all cases of OHCA in Sweden, allowing us to validate the model using data available to us. While we did try neural networks, random forest, and several other machine learning frameworks, we did not perform an exhaustive hyperparameter tuning. The selection of extreme gradient boosting was based on the fact that it outperformed all other models in the initial grid searches. A vast body of science has demonstrated this framework to outperform all others when modeling structured data generally. As can be seen in Table S3, there was a relatively high degree of missingness relating to some variables. By using multiple imputations and comparing imputed with original data, we conclude that the missing values were unlikely to impact the overall results.

### Conclusion

The SCARS prediction model can reliably and rapidly estimate the likelihood of survival using five to ten readily available variables such as ROSC, consciousness in the ED, use of adrenaline, initial presentation, and age. The model can provide clinicians with critical information during ongoing CPR in the ED.

## Contributors

All authors read and approved the final version of the manuscript. FH and ArR: Conceptualization, Data curation, Formal analysis, Funding acquisition, Methodology, Project administration, Supervision, Validation, Visualization, Writing - review & editing. JH; JE; PL: Conceptualization, Data curation, Formal analysis, Methodology, Project administration, Supervision, Validation, Visualization, Writing - review & editing. DLP, EO, AiR, EH, CD, HF, BR, NN, AF: Methodology, Visualization, Writing - review & editing. FH and ArR had full access to and verified the underlying data.

## Data sharing statement

Data sharing is available upon request and approval by the Swedish Ethical Review Board. The statistical code needed to reproduce the results in the article is available upon request.

## Declaration of interests

Johan Engdahl: received grants from Roche, The Stockholm Region, Carl Bennet AB, Swedish Heart-Lung Foundation, Swedish research Foundation, Swedish stroke foundation, Vinnova and consulting fees from Pfizer, Boehringer Ingelheim(BI), Bristol Myers Squibb (BMS), Philips, Piotrode, Merck Sharp and Dome and Honoraria from Pfizer,BMS, Philips, Merck Sharp and Dome and BI and participated in data monitoring board for Pfizer, BMS and Roche.

Deepak L Bhatt: Advisory Board: AngioWave, Bayer, Boehringer Ingelheim, Cardax, CellProthera, Cereno Scientific, Elsevier Practice Update Cardiology, High Enroll, Janssen, Level Ex, McKinsey, Medscape Cardiology, Merck, MyoKardia, NirvaMed, Novo Nordisk, PhaseBio, PLx Pharma, Regado Biosciences, Stasys; Board of Directors: AngioWave (stock options), Boston VA Research Institute, Bristol Myers Squibb (stock), DRS.LINQ (stock options), High Enroll (stock), Society of Cardiovascular Patient Care, TobeSoft; Chair: Inaugural Chair, American Heart Association Quality Oversight Committee; Consultant: Broadview Ventures; Data Monitoring Committees: Acesion Pharma, Assistance Publique-Hôpitaux de Paris, Baim Institute for Clinical Research (formerly Harvard Clinical Research Institute, for the PORTICO trial, funded by St. Jude Medical, now Abbott), Boston Scientific (Chair, PEITHO trial), Cleveland Clinic (including for the ExCEED trial, funded by Edwards), Contego Medical (Chair, PERFORMANCE 2), Duke Clinical Research Institute, Mayo Clinic, Mount Sinai School of Medicine (for the ENVISAGE trial, funded by Daiichi Sankyo; for the ABILITY-DM trial, funded by Concept Medical), Novartis, Population Health Research Institute; Rutgers University (for the NIH-funded MINT Trial); Honoraria: American College of Cardiology (Senior Associate Editor, Clinical Trials and News, ACC.org; Chair, ACC Accreditation Oversight Committee), Arnold and Porter law firm (work related to Sanofi/Bristol-Myers Squibb clopidogrel litigation), Baim Institute for Clinical Research (formerly Harvard Clinical Research Institute; RE-DUAL PCI clinical trial steering committee funded by Boehringer Ingelheim; AEGIS-II executive committee funded by CSL Behring), Belvoir Publications (Editor in Chief, Harvard Heart Letter), Canadian Medical and Surgical Knowledge Translation Research Group (clinical trial steering committees), Cowen and Company, Duke Clinical Research Institute (clinical trial steering committees, including for the PRONOUNCE trial, funded by Ferring Pharmaceuticals), HMP Global (Editor in Chief, Journal of Invasive Cardiology), Journal of the American College of Cardiology (Guest Editor; Associate Editor), K2P (Co-Chair, interdisciplinary curriculum), Level Ex, Medtelligence/ReachMD (CME steering committees), MJH Life Sciences, Oakstone CME (Course Director, Comprehensive Review of Interventional Cardiology), Piper Sandler, Population Health Research Institute (for the COMPASS operations committee, publications committee, steering committee, and USA national co-leader, funded by Bayer), Slack Publications (Chief Medical Editor, Cardiology Today's Intervention), Society of Cardiovascular Patient Care (Secretary/Treasurer), WebMD (CME steering committees), Wiley (steering committee); Other: Clinical Cardiology (Deputy Editor), NCDR-ACTION Registry Steering Committee (Chair), VA CART Research and Publications Committee (Chair); Patent: Sotagliflozin (named on a patent for sotagliflozin assigned to Brigham and Women's Hospital who assigned to Lexicon; neither I nor Brigham and Women's Hospital receive any income from this patent); Research Funding: Abbott, Acesion Pharma, Afimmune, Aker Biomarine, Amarin, Amgen, AstraZeneca, Bayer, Beren, Boehringer Ingelheim, Boston Scientific, Bristol-Myers Squibb, Cardax, CellProthera, Cereno Scientific, Chiesi, CinCor, CSL Behring, Eisai, Ethicon, Faraday Pharmaceuticals, Ferring Pharmaceuticals, Forest Laboratories, Fractyl, Garmin, HLS Therapeutics, Idorsia, Ironwood, Ischemix, Janssen, Javelin, Lexicon, Lilly, Medtronic, Merck, Moderna, MyoKardia, NirvaMed, Novartis, Novo Nordisk, Owkin, Pfizer, PhaseBio, PLx Pharma, Recardio, Regeneron, Reid Hoffman Foundation, Roche, Sanofi, Stasys, Synaptic, The Medicines Company, Youngene, 89Bio; Royalties: Elsevier (Editor, Braunwald's Heart Disease); Site Co-Investigator: Abbott, Biotronik, Boston Scientific, CSI, Endotronix, St. Jude Medical (now Abbott), Philips, SpectraWAVE, Svelte, Vascular Solutions; Trustee: American College of Cardiology; Unfunded Research: FlowCo, Takeda.

Hans Friberg: Scientific advisor TEQCool, Lund, Sweden, board member Swedish resuscitation council.

Araz Rawshani: Unrestricted grant from the Swedish research council(2019-02019), the 10.13039/501100003793Swedish Heart-Lung Foundation (20200261) and the Swedish state (ALFGBG-971482).

All other authors have nothing to declare.
