# Supplementary material for: Predicting survival and neurological outcome in out-of-hospital cardiac arrest using machine learning: the SCARS model
Source: eBioMedicine. 2023 Feb 9;89:104464. doi: 10.1016/j.ebiom.2023.104464 (PMC9945645; doi:10.1016/j.ebiom.2023.104464)
Supplement: Supplementary Material [file mmc1.docx]

Supplements

## Supplemental discussion S1. Description of the Swedish EMS system and hospital system

As described by Hasselqvist-Ax et al (NEJM 2015 doi: 10.1056/NEJMoa1405796.), in Sweden there are approximately 850 ambulance organizations serving 10 million citizens. There is a one-tiered EMS system in Sweden for responses to all medical emergencies: the EMS units that can respond to an OHCA can provide both basic level of life support as-well as advanced life support. Data on EMS units is included in the Swedish Cardiopulmonary Resuscitation Registry. Sweden has 15 dispatch centers, all of which are similar in their organization and emergency-call processing. The dispatcher uses a standard protocol with a specific questionnaire for the identified emergency. In cases of suspected cardiac arrest, an ambulance is dispatched, and dispatchers are instructed to offer the caller a chance to perform telephone-assisted CPR. In addition to the EMS system, some regions include a dual dispatch of fire fighters and police officers when available. In addition, there is growing number of regions in Sweden where lay volunteers are recruited and, using a mobile-phone positioning system, alerted and dispatched to the site of the OHCA.

Under special circumstances and if the assumed cause of the OHCA can be determined, patients may be triaged directly to a specialized hospital (ie traumatic CA transfer directly to a trauma center when available, acute coronary syndromes transferred to a hospital with cardiac catheterization capabilities, etc). However, the majority of OHCA are transported by the EMS to the nearest hospital, including both low, middle and high volume centers.

Supplemental table S1. Description of candidate predictors by category: Features included from the Swedish in- and out-patient registry and the Swedish Prescribed Drug Registry

International classification of diseases (ICD) version 10

| A00-B99 | Certain infectious and parasitic diseases |
| --- | --- |
| C00-D48 | Neoplasms |
| D50-D89 | Diseases of the blood and blood-forming organs and certain disorders involving the immune mechanism |
| E00-E90 | Endocrine, nutritional and metabolic diseases |
| F00-F99 | Mental and behavioural disorders |
| G00-G99 | Diseases of the nervous system |
| H00-H59 | Diseases of the eye and adnexa |
| H60-H95 | Diseases of the ear and mastoid process |
| I00-I99 | Diseases of the circulatory system |
| J00-J99 | Diseases of the respiratory system |
| K00−K93 | Diseases of the digestive system |
| L00−L99 | Diseases of the skin and subcutaneous tissue |
| M00−M99 | Diseases of the musculoskeletal system and connective tissue |
| N00−N99 | Diseases of the genitourinary system |
| O00−O99 | Pregnancy, childbirth and the puerperium |
| P00−P96 | Certain conditions originating in the perinatal period |
| Q00−Q99 | Congenital malformations, deformations and chromosomal abnormalities |
| R00−R99 | Symptoms, signs and abnormal clinical and laboratory findings, not elsewhere classified |
| S00−T98 | Injury, poisoning and certain other consequences of external causes |
| V01−Y98 | External causes of morbidity and mortality |
| Z00−Z99 | Factors influencing health status and contact with health services |
| U00-U99 | Codes for special purposes |

Anatomical, Therapeutic, Chemical classification (ATC) 25^TH^ edition

| A | Alimentary tract and metabolism |
| --- | --- |
| B | Blood and blood forming organs |
| C | Cardiovascular system |
| D | Dermatologicals |
| G | Genito urinary system and sex hormones |
| H | Systemic hormonal preparations, excl. sex hormones and insulins |
| J | Antiinfectives for systemic use |
| L | Antineoplastic and immunomodulating agents |
| M | Musculo-skeletal system |
| N | Nervous system |
| P | Antiparasitic products, insecticides and repellents |
| R | Respiratory system |
| S | Sensory organs |
| V | Various |

## Supplemental figure S1. Study Flow chart


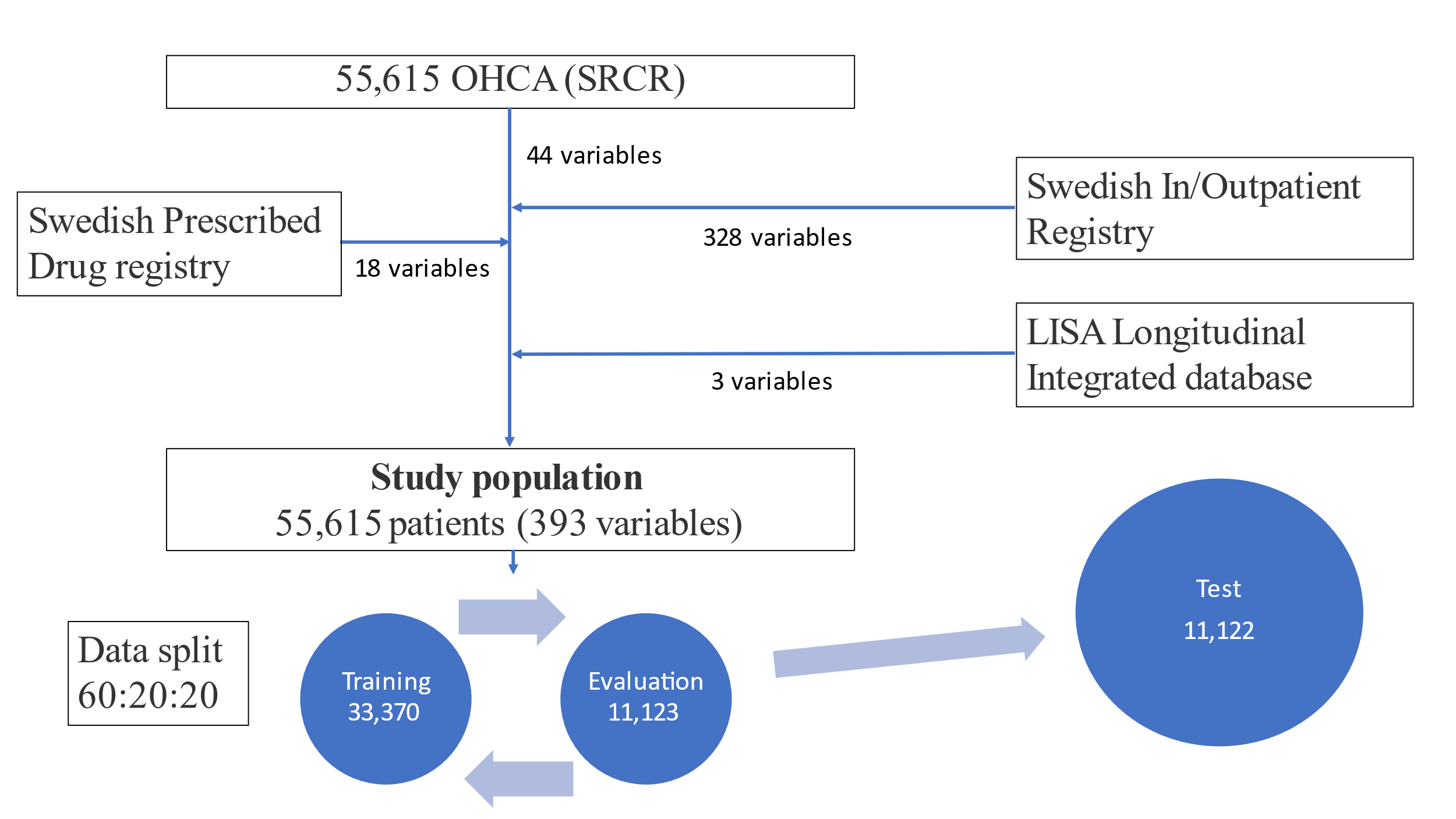


Figure legend Fig. S1: Figure S1 shows the flow chart and data source for patients included in the study. n= 55615. SRCR= Swedish Register for Cardiopulmonary Resuscitation.

## Supplemental figure S2. Hyperparameter tuning in extreme gradient boosting

Figure legend Fig. S2: Figure 2 shows the hyperparameter tuning in extreme gradient boosting using different learning rates (eta) and different subsamples with the number of boosting iterations on the x-axis and the ROC on the y-axis. Maximum tree depth is illustrated with red, green and blue dotted lines The Initial grid search showed that ROC was very high in all models, and the difference between them was very small. As evident, there were further room for improving ROC after the initial tuning, resulting in a second tuning in which no further improvement was observed. The best model had 1400 trees, maximum tree depth 10, shrinkage 0.01, gamma 0, column samples for each tree 0.8, minimum sum of instance weight needed in a child 1 and subsample ratio 0.7.

## Supplemental table S2. Grid search for hyperparameter tuning

|  | **nrounds** | **eta** | **max_depth** | **gamma** | **colsample_bytree** | **min_child_weight** | **subsample** |
| --- | --- | --- | --- | --- | --- | --- | --- |
| **1** | 800 | 0,01 | 8 | 0 | 0,8 | 1 | 0,7 |
| **2** | 900 | 0,01 | 8 | 0 | 0,8 | 1 | 0,7 |
| **3** | 1000 | 0,01 | 8 | 0 | 0,8 | 1 | 0,7 |
| **4** | 1100 | 0,01 | 8 | 0 | 0,8 | 1 | 0,7 |
| **5** | 1200 | 0,01 | 8 | 0 | 0,8 | 1 | 0,7 |
| **6** | 1300 | 0,01 | 8 | 0 | 0,8 | 1 | 0,7 |
| **7** | 1400 | 0,01 | 8 | 0 | 0,8 | 1 | 0,7 |
| **8** | 800 | 0,01 | 10 | 0 | 0,8 | 1 | 0,7 |
| **9** | 900 | 0,01 | 10 | 0 | 0,8 | 1 | 0,7 |
| **10** | 1000 | 0,01 | 10 | 0 | 0,8 | 1 | 0,7 |
| **11** | 1100 | 0,01 | 10 | 0 | 0,8 | 1 | 0,7 |
| **12** | 1200 | 0,01 | 10 | 0 | 0,8 | 1 | 0,7 |
| **13** | 1300 | 0,01 | 10 | 0 | 0,8 | 1 | 0,7 |
| **14** | 1400 | 0,01 | 10 | 0 | 0,8 | 1 | 0,7 |
| **15** | 200 | 0,01 | 6 | 0 | 0,8 | 1 | 0,5 |
| **16** | 300 | 0,01 | 6 | 0 | 0,8 | 1 | 0,5 |
| **17** | 400 | 0,01 | 6 | 0 | 0,8 | 1 | 0,5 |
| **18** | 500 | 0,01 | 6 | 0 | 0,8 | 1 | 0,5 |
| **19** | 600 | 0,01 | 6 | 0 | 0,8 | 1 | 0,5 |
| **20** | 700 | 0,01 | 6 | 0 | 0,8 | 1 | 0,5 |
| **21** | 800 | 0,01 | 6 | 0 | 0,8 | 1 | 0,5 |
| **22** | 900 | 0,01 | 6 | 0 | 0,8 | 1 | 0,5 |
| **23** | 200 | 0,05 | 6 | 0 | 0,8 | 1 | 0,5 |
| **24** | 300 | 0,05 | 6 | 0 | 0,8 | 1 | 0,5 |
| **25** | 400 | 0,05 | 6 | 0 | 0,8 | 1 | 0,5 |
| **26** | 500 | 0,05 | 6 | 0 | 0,8 | 1 | 0,5 |
| **27** | 600 | 0,05 | 6 | 0 | 0,8 | 1 | 0,5 |
| **28** | 700 | 0,05 | 6 | 0 | 0,8 | 1 | 0,5 |
| **29** | 800 | 0,05 | 6 | 0 | 0,8 | 1 | 0,5 |
| **30** | 900 | 0,05 | 6 | 0 | 0,8 | 1 | 0,5 |
| **31** | 200 | 0,01 | 8 | 0 | 0,8 | 1 | 0,5 |
| **32** | 300 | 0,01 | 8 | 0 | 0,8 | 1 | 0,5 |
| **33** | 400 | 0,01 | 8 | 0 | 0,8 | 1 | 0,5 |
| **34** | 500 | 0,01 | 8 | 0 | 0,8 | 1 | 0,5 |
| **35** | 600 | 0,01 | 8 | 0 | 0,8 | 1 | 0,5 |
| **36** | 700 | 0,01 | 8 | 0 | 0,8 | 1 | 0,5 |
| **37** | 800 | 0,01 | 8 | 0 | 0,8 | 1 | 0,5 |
| **38** | 900 | 0,01 | 8 | 0 | 0,8 | 1 | 0,5 |
| **39** | 200 | 0,05 | 8 | 0 | 0,8 | 1 | 0,5 |
| **40** | 300 | 0,05 | 8 | 0 | 0,8 | 1 | 0,5 |
| **41** | 400 | 0,05 | 8 | 0 | 0,8 | 1 | 0,5 |
| **42** | 500 | 0,05 | 8 | 0 | 0,8 | 1 | 0,5 |
| **43** | 600 | 0,05 | 8 | 0 | 0,8 | 1 | 0,5 |
| **44** | 700 | 0,05 | 8 | 0 | 0,8 | 1 | 0,5 |
| **45** | 800 | 0,05 | 8 | 0 | 0,8 | 1 | 0,5 |
| **46** | 900 | 0,05 | 8 | 0 | 0,8 | 1 | 0,5 |
| **47** | 200 | 0,01 | 10 | 0 | 0,8 | 1 | 0,5 |
| **48** | 300 | 0,01 | 10 | 0 | 0,8 | 1 | 0,5 |
| **49** | 400 | 0,01 | 10 | 0 | 0,8 | 1 | 0,5 |
| **50** | 500 | 0,01 | 10 | 0 | 0,8 | 1 | 0,5 |
| **51** | 600 | 0,01 | 10 | 0 | 0,8 | 1 | 0,5 |
| **52** | 700 | 0,01 | 10 | 0 | 0,8 | 1 | 0,5 |
| **53** | 800 | 0,01 | 10 | 0 | 0,8 | 1 | 0,5 |
| **54** | 900 | 0,01 | 10 | 0 | 0,8 | 1 | 0,5 |
| **55** | 200 | 0,05 | 10 | 0 | 0,8 | 1 | 0,5 |
| **56** | 300 | 0,05 | 10 | 0 | 0,8 | 1 | 0,5 |
| **57** | 400 | 0,05 | 10 | 0 | 0,8 | 1 | 0,5 |
| **58** | 500 | 0,05 | 10 | 0 | 0,8 | 1 | 0,5 |
| **59** | 600 | 0,05 | 10 | 0 | 0,8 | 1 | 0,5 |
| **60** | 700 | 0,05 | 10 | 0 | 0,8 | 1 | 0,5 |
| **61** | 800 | 0,05 | 10 | 0 | 0,8 | 1 | 0,5 |
| **62** | 900 | 0,05 | 10 | 0 | 0,8 | 1 | 0,5 |
| **63** | 200 | 0,01 | 6 | 0 | 0,8 | 1 | 0,7 |
| **64** | 300 | 0,01 | 6 | 0 | 0,8 | 1 | 0,7 |
| **65** | 400 | 0,01 | 6 | 0 | 0,8 | 1 | 0,7 |
| **66** | 500 | 0,01 | 6 | 0 | 0,8 | 1 | 0,7 |
| **67** | 600 | 0,01 | 6 | 0 | 0,8 | 1 | 0,7 |
| **68** | 700 | 0,01 | 6 | 0 | 0,8 | 1 | 0,7 |
| **69** | 800 | 0,01 | 6 | 0 | 0,8 | 1 | 0,7 |
| **70** | 900 | 0,01 | 6 | 0 | 0,8 | 1 | 0,7 |
| **71** | 200 | 0,05 | 6 | 0 | 0,8 | 1 | 0,7 |
| **72** | 300 | 0,05 | 6 | 0 | 0,8 | 1 | 0,7 |
| **73** | 400 | 0,05 | 6 | 0 | 0,8 | 1 | 0,7 |
| **74** | 500 | 0,05 | 6 | 0 | 0,8 | 1 | 0,7 |
| **75** | 600 | 0,05 | 6 | 0 | 0,8 | 1 | 0,7 |
| **76** | 700 | 0,05 | 6 | 0 | 0,8 | 1 | 0,7 |
| **77** | 800 | 0,05 | 6 | 0 | 0,8 | 1 | 0,7 |
| **78** | 900 | 0,05 | 6 | 0 | 0,8 | 1 | 0,7 |
| **79** | 200 | 0,01 | 8 | 0 | 0,8 | 1 | 0,7 |
| **80** | 300 | 0,01 | 8 | 0 | 0,8 | 1 | 0,7 |
| **81** | 400 | 0,01 | 8 | 0 | 0,8 | 1 | 0,7 |
| **82** | 500 | 0,01 | 8 | 0 | 0,8 | 1 | 0,7 |
| **83** | 600 | 0,01 | 8 | 0 | 0,8 | 1 | 0,7 |
| **84** | 700 | 0,01 | 8 | 0 | 0,8 | 1 | 0,7 |
| **85** | 800 | 0,01 | 8 | 0 | 0,8 | 1 | 0,7 |
| **86** | 900 | 0,01 | 8 | 0 | 0,8 | 1 | 0,7 |
| **87** | 200 | 0,05 | 8 | 0 | 0,8 | 1 | 0,7 |
| **88** | 300 | 0,05 | 8 | 0 | 0,8 | 1 | 0,7 |
| **89** | 400 | 0,05 | 8 | 0 | 0,8 | 1 | 0,7 |
| **90** | 500 | 0,05 | 8 | 0 | 0,8 | 1 | 0,7 |
| **91** | 600 | 0,05 | 8 | 0 | 0,8 | 1 | 0,7 |
| **92** | 700 | 0,05 | 8 | 0 | 0,8 | 1 | 0,7 |
| **93** | 800 | 0,05 | 8 | 0 | 0,8 | 1 | 0,7 |
| **94** | 900 | 0,05 | 8 | 0 | 0,8 | 1 | 0,7 |
| **95** | 200 | 0,01 | 10 | 0 | 0,8 | 1 | 0,7 |
| **96** | 300 | 0,01 | 10 | 0 | 0,8 | 1 | 0,7 |
| **97** | 400 | 0,01 | 10 | 0 | 0,8 | 1 | 0,7 |
| **98** | 500 | 0,01 | 10 | 0 | 0,8 | 1 | 0,7 |
| **99** | 600 | 0,01 | 10 | 0 | 0,8 | 1 | 0,7 |
| **100** | 700 | 0,01 | 10 | 0 | 0,8 | 1 | 0,7 |
| **101** | 800 | 0,01 | 10 | 0 | 0,8 | 1 | 0,7 |
| **102** | 900 | 0,01 | 10 | 0 | 0,8 | 1 | 0,7 |
| **103** | 200 | 0,05 | 10 | 0 | 0,8 | 1 | 0,7 |
| **104** | 300 | 0,05 | 10 | 0 | 0,8 | 1 | 0,7 |
| **105** | 400 | 0,05 | 10 | 0 | 0,8 | 1 | 0,7 |
| **106** | 500 | 0,05 | 10 | 0 | 0,8 | 1 | 0,7 |
| **107** | 600 | 0,05 | 10 | 0 | 0,8 | 1 | 0,7 |
| **108** | 700 | 0,05 | 10 | 0 | 0,8 | 1 | 0,7 |
| **109** | 800 | 0,05 | 10 | 0 | 0,8 | 1 | 0,7 |
| **110** | 900 | 0,05 | 10 | 0 | 0,8 | 1 | 0,7 |
| **111** | 200 | 0,01 | 6 | 0 | 0,8 | 1 | 0,8 |
| **112** | 300 | 0,01 | 6 | 0 | 0,8 | 1 | 0,8 |
| **113** | 400 | 0,01 | 6 | 0 | 0,8 | 1 | 0,8 |
| **114** | 500 | 0,01 | 6 | 0 | 0,8 | 1 | 0,8 |
| **115** | 600 | 0,01 | 6 | 0 | 0,8 | 1 | 0,8 |
| **116** | 700 | 0,01 | 6 | 0 | 0,8 | 1 | 0,8 |
| **117** | 800 | 0,01 | 6 | 0 | 0,8 | 1 | 0,8 |
| **118** | 900 | 0,01 | 6 | 0 | 0,8 | 1 | 0,8 |
| **119** | 200 | 0,05 | 6 | 0 | 0,8 | 1 | 0,8 |
| **120** | 300 | 0,05 | 6 | 0 | 0,8 | 1 | 0,8 |
| **121** | 400 | 0,05 | 6 | 0 | 0,8 | 1 | 0,8 |
| **122** | 500 | 0,05 | 6 | 0 | 0,8 | 1 | 0,8 |
| **123** | 600 | 0,05 | 6 | 0 | 0,8 | 1 | 0,8 |
| **124** | 700 | 0,05 | 6 | 0 | 0,8 | 1 | 0,8 |
| **125** | 800 | 0,05 | 6 | 0 | 0,8 | 1 | 0,8 |
| **126** | 900 | 0,05 | 6 | 0 | 0,8 | 1 | 0,8 |
| **127** | 200 | 0,01 | 8 | 0 | 0,8 | 1 | 0,8 |
| **128** | 300 | 0,01 | 8 | 0 | 0,8 | 1 | 0,8 |
| **129** | 400 | 0,01 | 8 | 0 | 0,8 | 1 | 0,8 |
| **130** | 500 | 0,01 | 8 | 0 | 0,8 | 1 | 0,8 |
| **131** | 600 | 0,01 | 8 | 0 | 0,8 | 1 | 0,8 |
| **132** | 700 | 0,01 | 8 | 0 | 0,8 | 1 | 0,8 |
| **133** | 800 | 0,01 | 8 | 0 | 0,8 | 1 | 0,8 |
| **134** | 900 | 0,01 | 8 | 0 | 0,8 | 1 | 0,8 |
| **135** | 200 | 0,05 | 8 | 0 | 0,8 | 1 | 0,8 |
| **136** | 300 | 0,05 | 8 | 0 | 0,8 | 1 | 0,8 |
| **137** | 400 | 0,05 | 8 | 0 | 0,8 | 1 | 0,8 |
| **138** | 500 | 0,05 | 8 | 0 | 0,8 | 1 | 0,8 |
| **139** | 600 | 0,05 | 8 | 0 | 0,8 | 1 | 0,8 |
| **140** | 700 | 0,05 | 8 | 0 | 0,8 | 1 | 0,8 |
| **141** | 800 | 0,05 | 8 | 0 | 0,8 | 1 | 0,8 |
| **142** | 900 | 0,05 | 8 | 0 | 0,8 | 1 | 0,8 |
| **143** | 200 | 0,01 | 10 | 0 | 0,8 | 1 | 0,8 |
| **144** | 300 | 0,01 | 10 | 0 | 0,8 | 1 | 0,8 |
| **145** | 400 | 0,01 | 10 | 0 | 0,8 | 1 | 0,8 |
| **146** | 500 | 0,01 | 10 | 0 | 0,8 | 1 | 0,8 |
| **147** | 600 | 0,01 | 10 | 0 | 0,8 | 1 | 0,8 |
| **148** | 700 | 0,01 | 10 | 0 | 0,8 | 1 | 0,8 |
| **149** | 800 | 0,01 | 10 | 0 | 0,8 | 1 | 0,8 |
| **150** | 900 | 0,01 | 10 | 0 | 0,8 | 1 | 0,8 |
| **151** | 200 | 0,05 | 10 | 0 | 0,8 | 1 | 0,8 |
| **152** | 300 | 0,05 | 10 | 0 | 0,8 | 1 | 0,8 |
| **153** | 400 | 0,05 | 10 | 0 | 0,8 | 1 | 0,8 |
| **154** | 500 | 0,05 | 10 | 0 | 0,8 | 1 | 0,8 |
| **155** | 600 | 0,05 | 10 | 0 | 0,8 | 1 | 0,8 |
| **156** | 700 | 0,05 | 10 | 0 | 0,8 | 1 | 0,8 |
| **157** | 800 | 0,05 | 10 | 0 | 0,8 | 1 | 0,8 |
| **158** | 900 | 0,05 | 10 | 0 | 0,8 | 1 | 0,8 |

## Supplemental table S3. Missingness (non-imputed data) for the top 20 predictors in the final model

| **Variable** | **missing (n)** | **(%)** | **Missing expected** |
| --- | --- | --- | --- |
| Return of spontaneous circulation at hospital arrival | 23869 | 43 | No |
| Patient breathing at EMS arrival at scene | 24526 | 44 | No |
| Adrenalin | 678 | 1 | No |
| Age yr (mean) ((SD)) | 0 | 0 | No |
| Initial Rhythm | 6572 | 12 | No |
| Time from collapse to EMS arrival | 19959 | 36 | Yes, when time of collapse uncertain |
| Collapse to EMS dispatch | 23580 | 42 | Yes, when time of collapse uncertain |
| Time from EMS dispatch to EMS arrival | 7278 | 13 | Yes, when time of collapse uncertain |
| Collapse to CPR | 12164 | 22 | Yes, when time of collapse uncertain |
| Time to ROSC | 40928 | 74 | Yes, if ROSC did not occurr |
| Year | 0 | 0 | No |
| Breathing | 1583 | 3 | No |
| Laryngeal Mask | 22898 | 41 | No |
| Endotracheal intubation | 988 | 2 | No |
| Bystander education | 44120 | 79 | No |
| Collapse to first defibrillation | 2053 | 18 | Yes, if not defibrillated |
| Number of defibrillations | 813 | 7 | Yes, if not defibrillated |

## Supplemental figure S3. Model evaluation on training data and ROC curve on test data for CPC-score

Figure legend Fig. S3: Figure S3 shows model evaluation on training data and performance (ROC-curve) on test data regarding CPC-score.

## Supplemental figure S4. SCARS-1 web application with 3 example patients

1. Navigate to <https://arara1.shinyapps.io/scaars/> in any web browser.


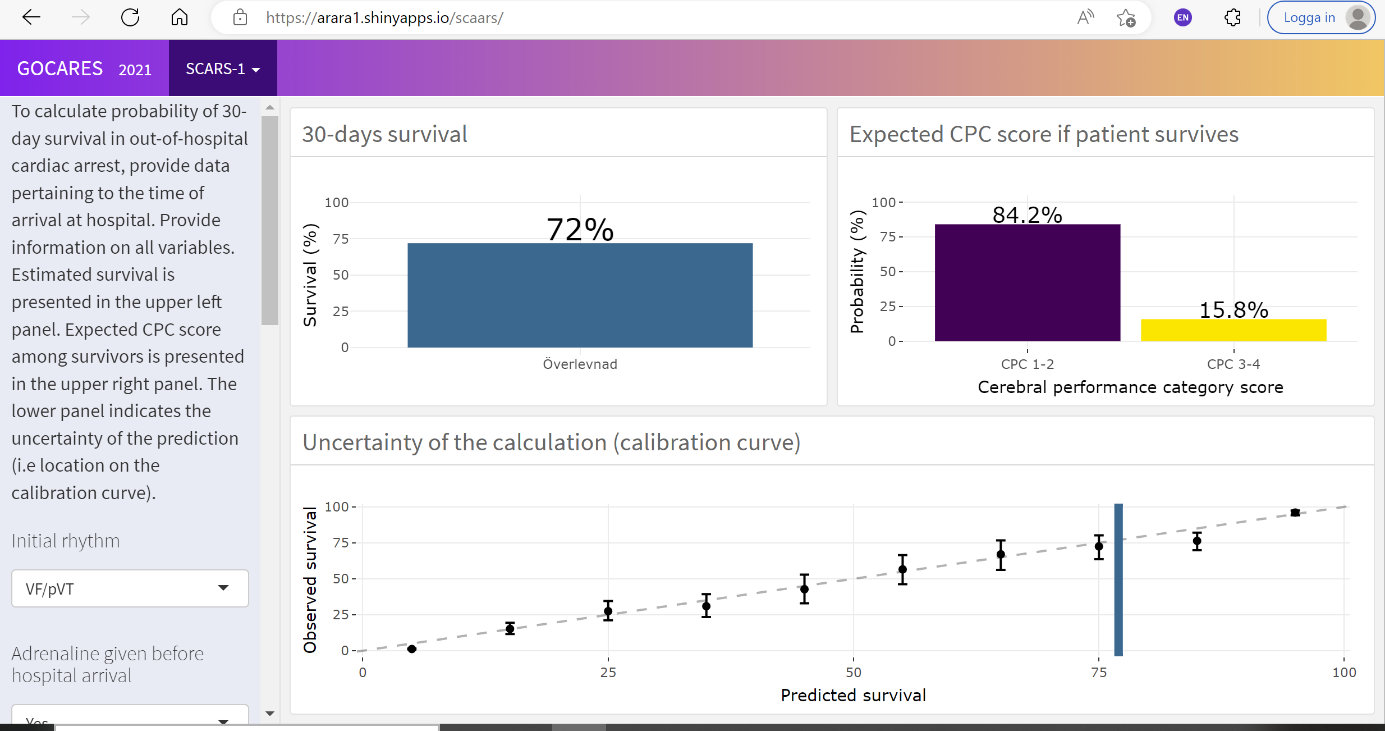


1. Enter 8 variables regarding the circumstances at resuscitation on the left of the screen
   1. Initial rhythm
   2. Adrenaline given before hospital arrival
   3. Consciousness on hospital arrival
   4. ROSC on hospital arrival
   5. Age
   6. Time to start of CPR
   7. Time to EMS arrival
   8. Number of defibrillations
2. Time of day and calendar year are included automatically
3. The upper left figure shows the estimated chance of 30-day survival


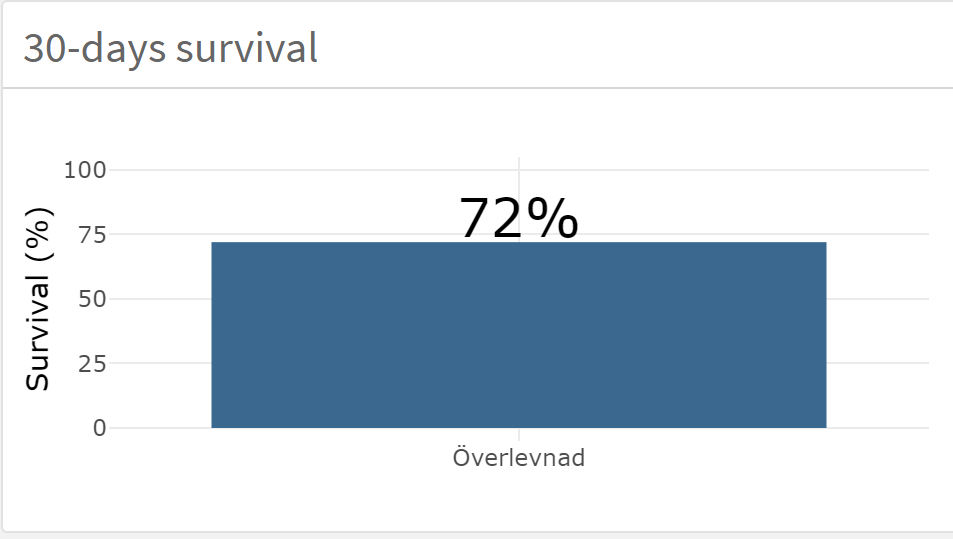


1. The upper right figure shows the estimated CPC-score at hospital discharge if the patient survives.


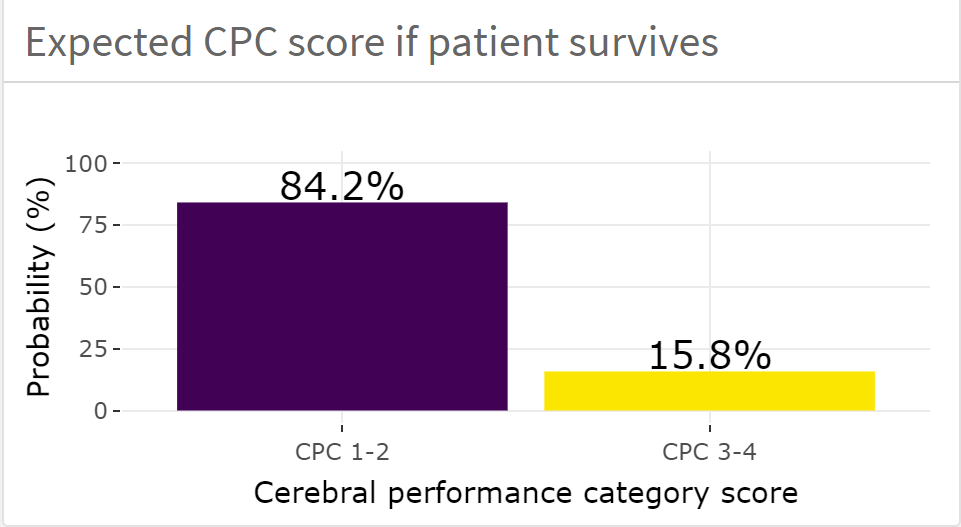


1. The lower figure shows the Calibration curve for the survival estimation, ie the uncertainty of the estimation.


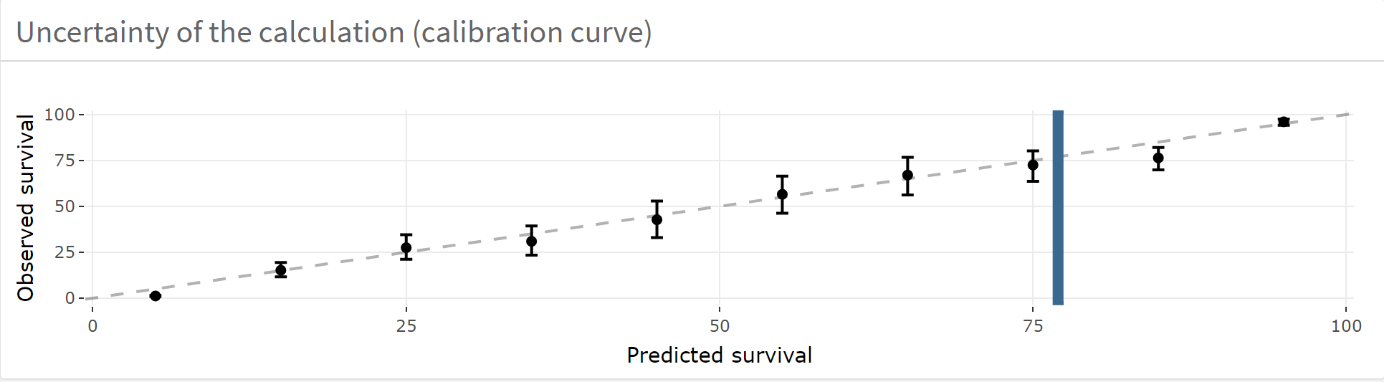


1. By altering the values to the left of the screen, the estimation of survival, the CPC-score and the calibration curve changes.

3 patient examples

Patient 1: 80 yo with ventricular fibrillation as initial rhythm, 4 minutes from CA to start of CPR, 10 minutes from CA to EMS arrival, no adrenaline given, one defibrillation, ROSC on hospital admission but unconscious. The SCARS-1 application estimates that this patients has a 93 % chance of 30-day survival and a 84% likelihood of of a good neurological outcome at hospital discharge (CPC 1-2) if he or she survives. The calibration curve illustrates that the estimation has a low level of uncertainty.


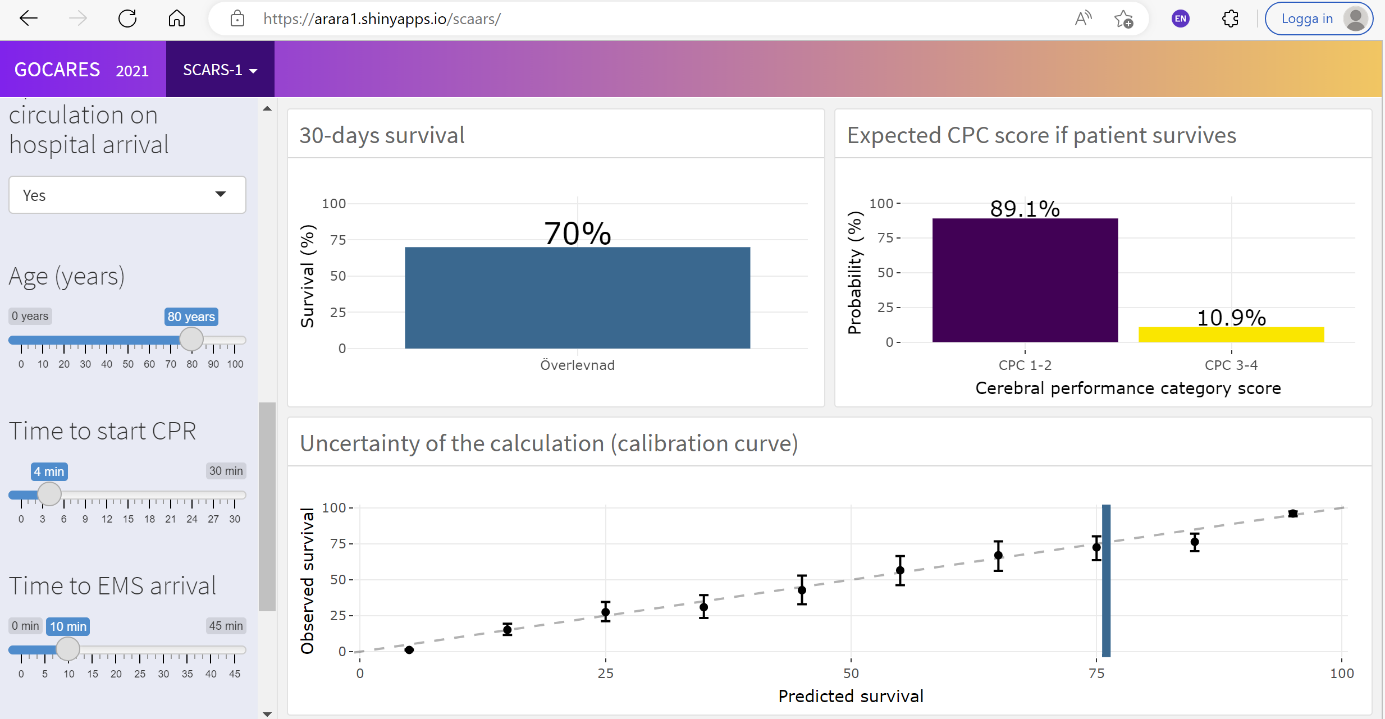


Patient 2: 90 yo with asystole as initial rhythm, 0 minutes from CA to start of CPR, 10 minutes from CA to EMS arrival, adrenaline given, no defibrillation, no ROSC on hospital admission and still unconscious. The SCARS-1 application estimates that this patients has a 1 % chance of 30-day survival and a 67,7 % likelihood of a good neurological outcome at hospital discharge (CPC 1-2) in case of survival. The calibration curve illustrates that the estimation has a low level of uncertainty.


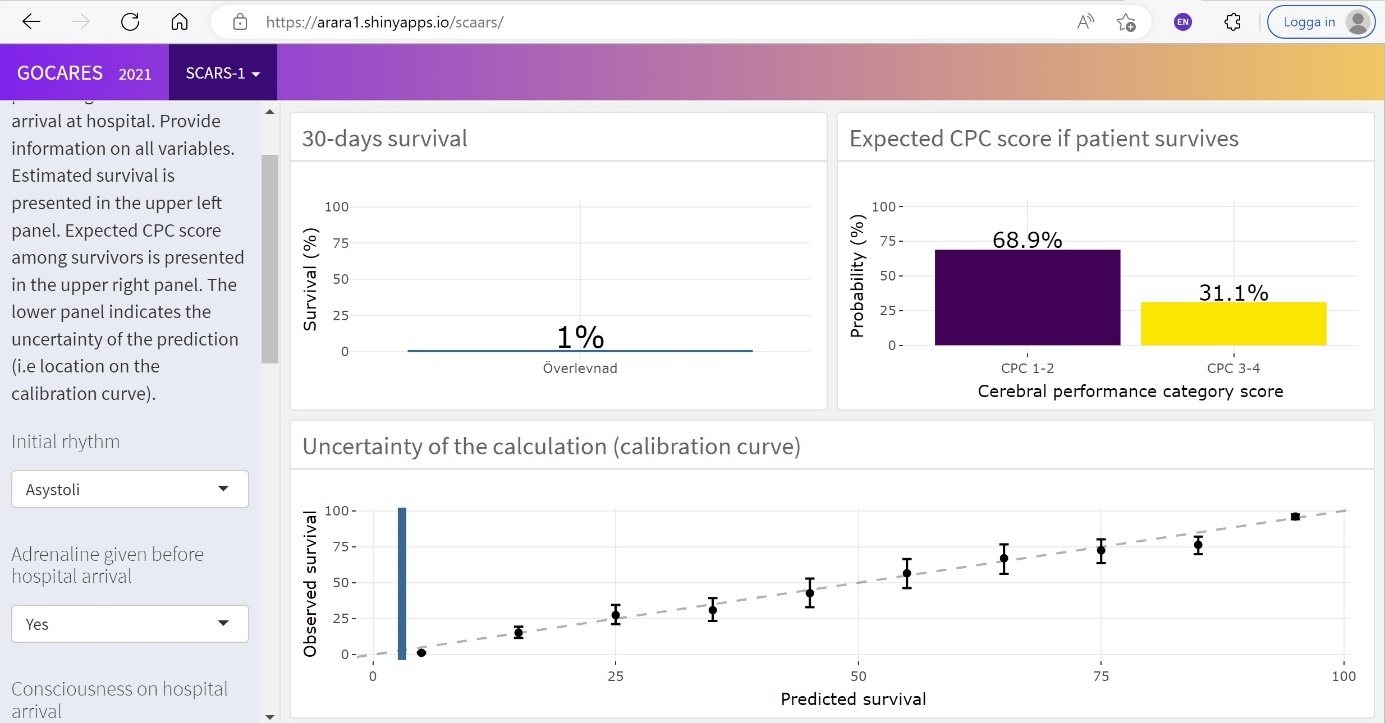


Patient 3: 30 yo with PEA as initial rhythm, 5 minutes from CA to start of CPR, 5 minutes from CA to EMS arrival, adrenaline given, 3 defibrillations, ROSC on hospital admission and still unconscious. The SCARS-1 application estimates that patients has a 45 % chance of 30-day survival and a 80.0 % likelihood of a good neurological outcome at hospital discharge (CPC 1-2) in case of survival. The calibration curve illustrates that the estimation has a low level of uncertainty.


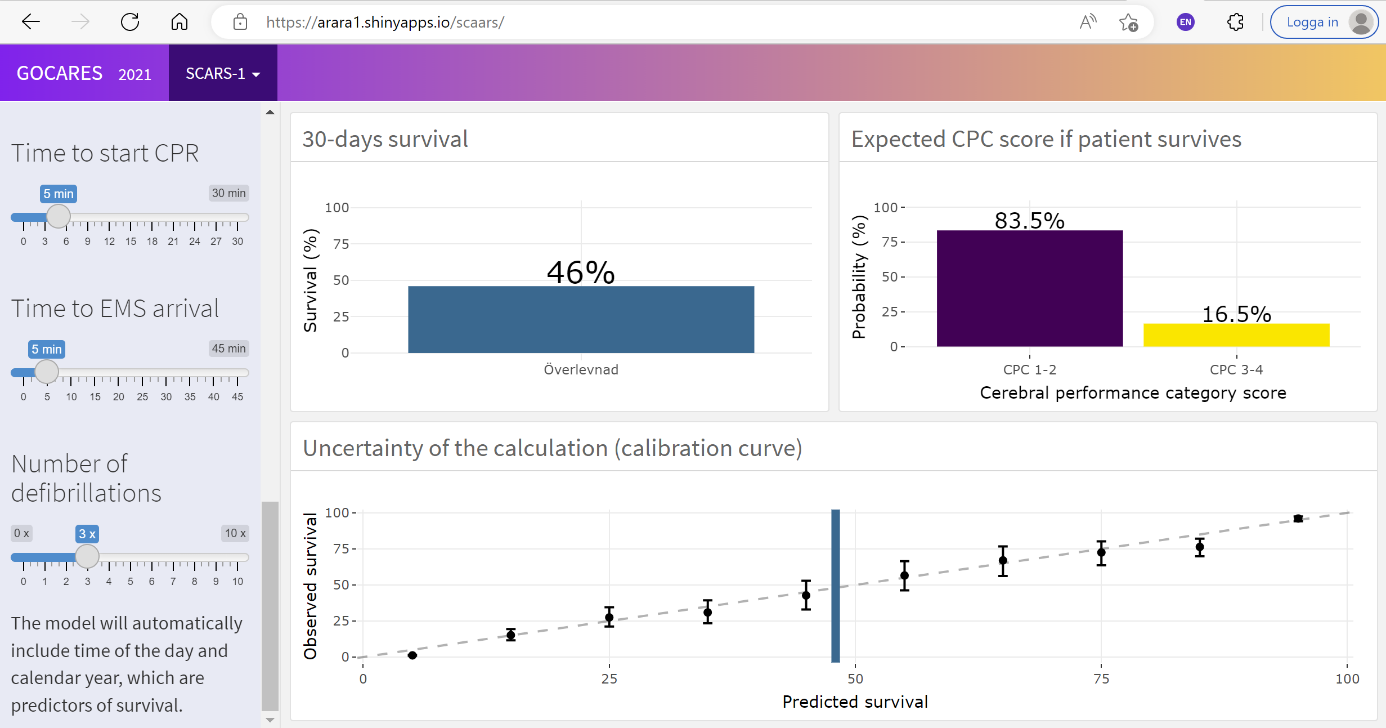


## Supplemental table S4. Baseline characteristics of 55,615 patients with out of hospital cardiac arrest stratified by survival at 30 days

| Table S4. Baseline characteristics of 55,615 patients with out of hospital cardiac arrest in relation to 30-days survival | | |
| --- | --- | --- |
| **Characteristic** | Alive | Dead |
| Patients, n | 6191 | 49424 |
| Female Sex, n (%) | 1548 (25.0) | 17333 (35.1) |
| Age yr (mean) ((SD)) | 61.25 (18.75) | 69.83 (17.58) |
| **Cause of OHCA** **– n (%)** |  |  |
| Cardiac | 3904 (70.9) | 26912 (61.4) |
| Overdose | 286 (5.2) | 1141 (2.6) |
| Accident | 57 (1.0) | 1082 (2.5) |
| Pulmonary disease | 140 (2.5) | 2625 (6.0) |
| Asphyxia | 116 (2.1) | 1170 (2.7) |
| Suicide* | 48 (0.9) | 1073 (2.4) |
| Drowning | 99 (1.8) | 366 (0.8) |
| Sudden infant death | 13 (0.2) | 157 (0.4) |
| Other | 844 (15.3) | 9311 (21.2) |
| **Location of cardiac arrest – n (%)** |  |  |
| Home | 2844 (46.2) | 36817 (74.8) |
| Public location | 2037 (33.1) | 6952 (14.1) |
| Other locations | 1275 (20.7) | 5453 (11.1) |
| **Critical time intervals, minutes – median (IQR)** |  |  |
| Collapse to EMS dispatch | 2.00 [1.00, 3.00] | 2.00 [1.00, 6.00] |
| Collapse to CPR | 1.00 [0.00, 3.00] | 3.00 [0.00, 10.00] |
| Collapse to first defibrillation | 8.00 [2.00, 13.00] | 17.00 [11.00, 26.00] |
| Time from collapse to EMS arrival | 9.00 [6.00, 15.00] | 13.00 [8.00, 20.00] |
| Time from EMS dispatch to EMS arrival | 8.00 [5.00, 14.00] | 11.00 [7.00, 17.00] |
| Time to ROSC | 12.00 [6.00, 19.00] | 15.00 [10.00, 24.00] |
| **Circumstance at time of CA – n (%)** |  |  |
| Witnessed CA | 5267 (87.7) | 29656 (61.8) |
| CA witnessed by EMS | 830 (29.0) | 3086 (19.9) |
| Telephone CPR | 1009 (54.0) | 8556 (64.0) |
| Training CPR provider |  |  |
| Layperson without CPR training | 507 (36.9) | 4882 (48.2) |
| Layperson with CPR training | 504 (36.7) | 3711 (36.7) |
| Health care worker | 362 (26.4) | 1529 (15.1) |
| **Initial presentation et EMS arrival – n (%)** |  |  |
| **Initial Rhythm** |  |  |
| VF/pVT, | 3741 (80.0) | 7633 (17.2) |
| PEA, | 432 (9.2) | 7989 (18.0) |
| Asystole | 503 (10.8) | 28745 (64.8) |
| Patient conscious at EMS arrival at scene | 1748 (28.8) | 3980 (8.3) |
| Patient breathing at EMS arrival at scene |  |  |
| No breathing | 2355 (39.0) | 39652 (82.6) |
| Agonal breathing | 1564 (25.9) | 4227 (8.8) |
| Normal breathing, | 2109 (35.0) | 4103 (8.5) |
| Unknown, | 4 (0.1) | 18 (0.0) |
| Pulse at EMS arrival at scene | 2340 (40.2) | 5044 (10.7) |
| Return of spontaneaus circulation at hospital arrival | 5485 (94.8) | 8705 (33.5) |
| Conscious at hospital arrival | 2608 (45.8) | 810 (3.2) |
| **Prehospital interventions – n (%)** |  |  |
| Bystander treatment before EMS arrival - Yes | 3611 (61.2) | 25880 (54.3) |
| CPR before EMS arrival -Yes | 1554 (59.9) | 11934 (57.0) |
| Public AED attached to patient – Yes | 456 (14.6) | 1407 (5.6) |
| Bystander def. with public AED – Yes | 320 (71.7) | 332 (24.4) |
| Mechanical CPR | 1412 (23.9) | 20074 (42.4) |
| Ventilation | 4939 (80.7) | 46203 (94.1) |
| Endotracheal intubation | 1017 (16.8) | 14420 (29.7) |
| Laryngeal Mask | 1241 (34.1) | 18719 (64.4) |
| Defibrillation | 4013 (66.4) | 13799 (29.1) |
| Number of defibrillations, n (mean (SD)) | 2.68 (2.30) | 3.71 (3.34) |
| Adrenalin | 2160 (35.8) | 41130 (84.1) |
| Amiodarone | 932 (15.6) | 5433 (11.3) |
| **Coexisting conditions,** n (%) |  |  |
| Hypertension, | 2004 (32.4) | 22817 (46.2) |
| Fall associated with the CA | 1691 (27.3) | 17632 (35.7) |
| Heart failure | 953 (15.4) | 11716 (23.7) |
| Ischemic heart disease | 1143 (18.5) | 10362 (21.0) |
| Atrial flutter/fibrillation | 923 (14.9) | 10440 (21.1) |
| Type 2 Diabetes Mellitus | 722 (11.7) | 9887 (20.0) |
| Arthrosis | 802 (13.0) | 7908 (16.0) |
| Hyperlipidemia | 849 (13.7) | 7850 (15.9) |
| Trauma to the head associated with the CA | 916 (14.8) | 7590 (15.4) |
| Angina pectoris | 825 (13.3) | 7667 (15.5) |
| Epilepsy | 795 (12.8) | 7555 (15.3) |
| Malignancy | 617 (10.0) | 7324 (14.8) |
| **Medications used prior to OHCA, n (%)** |  |  |
| Anticoagulants | 1650 (26.7) | 18476 (37.4) |
| Beta blockers | 1593 (25.7) | 17047 (34.5) |
| ACE-inhibitors | 1785 (28.8) | 16414 (33.2) |
| Diuretics | 947 (15.3) | 14073 (28.5) |
| Hyperlipidemia medications | 1275 (20.6) | 11946 (24.2) |
| Antiacids | 728 (11.8) | 10415 (21.1) |
| Calcium channel blockers | 814 (13.1) | 8024 (16.2) |
| Diabetes medications | 576 (9.3) | 7724 (15.6) |
| Antibiotics | 469 (7.6) | 7261 (14.7) |
| Antiarrhytmics | 496 (8.0) | 6120 (12.4) |
| Horomone modulators | 152 (2.5) | 1338 (2.7) |
| Other hypertension medications | 47 (0.8) | 585 (1.2) |

## Supplemental figure S5. Crude rates of ROSC, hospitalization and 30-days survival

Figure legend Fig. S5: Figure S5 shows crude rates of ROSC, hospitalization and 30-day survival by cause of OHCA. N=55615. SIDS= sudden infant death syndrome

## Supplemental table S5. Tuning the decision threshold (cut-off) evaluation data set

**1. Default threshold: 50%**

Confusion Matrix and Statistics

| prediction | Alive | Dead |
| --- | --- | --- |
| Alive | 956 | 155 |
| Dead | 282 | 9730 |

Accuracy : 0.9607

95% CI : (0.9569, 0.9642)

No Information Rate : 0.8887

P-Value [Acc > NIR] : < 2.2e-16

Kappa : 0.7921

Mcnemar's Test P-Value : 1.666e-09

Sensitivity : 0.77221

Specificity : 0.98432

Pos Pred Value : 0.86049

Neg Pred Value : 0.97183

Prevalence : 0.11130

Detection Rate : 0.08595

Detection Prevalence : 0.09988

Balanced Accuracy : 0.87827

**2. Maximizing Youden index**

Confusion Matrix and Statistics

| prediction | Alive | Dead |
| --- | --- | --- |
| Alive | 1152 | 760 |
| Dead | 86 | 9125 |

Accuracy : 0.9239

95% CI : (0.9189, 0.9288)

No Information Rate : 0.8887

P-Value [Acc > NIR] : < 2.2e-16

Kappa : 0.6895

Mcnemar's Test P-Value : < 2.2e-16

Sensitivity : 0.9305

Specificity : 0.9231

Pos Pred Value : 0.6025

Neg Pred Value : 0.9907

Prevalence : 0.1113

Detection Rate : 0.1036

Detection Prevalence : 0.1719

Balanced Accuracy : 0.9268

**3. Achieving 95% sensitivity**

Confusion Matrix and Statistics

| prediction | Alive | Dead |
| --- | --- | --- |
| Alive | 1177 | 1079 |
| Dead | 61 | 8806 |

Accuracy : 0.8975

95% CI : (0.8917, 0.9031)

No Information Rate : 0.8887

P-Value [Acc > NIR] : 0.001487

Kappa : 0.619

Mcnemar's Test P-Value : < 2.2e-16

Sensitivity : 0.9507

Specificity : 0.8908

Pos Pred Value : 0.5217

Neg Pred Value : 0.9931

Prevalence : 0.1113

Detection Rate : 0.1058

Detection Prevalence : 0.2028

Balanced Accuracy : 0.9208

## Supplemental table S6. Model performance on test data

**1. Default threshold: 50%**

Confusion Matrix and Statistics

| prediction | Alive | Dead |
| --- | --- | --- |
| Alive | 943 | 177 |
| Dead | 295 | 9707 |

Accuracy : 0.9576

95% CI : (0.9536, 0.9612)

No Information Rate : 0.8887

P-Value [Acc > NIR] : < 2.2e-16

Kappa : 0.7762

Mcnemar's Test P-Value : 7.23e-08

Sensitivity : 0.76171

Specificity : 0.98209

Pos Pred Value : 0.84196

Neg Pred Value : 0.97051

Prevalence : 0.11131

Detection Rate : 0.08479

Detection Prevalence : 0.10070

Balanced Accuracy : 0.87190

**2. Maximizing Youden index**

Confusion Matrix and Statistics

| prediction | Alive | Dead |
| --- | --- | --- |
| Alive | 1134 | 818 |
| Dead | 104 | 9066 |

Accuracy : 0.9171

95% CI : (0.9118, 0.9222)

No Information Rate : 0.8887

P-Value [Acc > NIR] : < 2.2e-16

Kappa : 0.6654

Mcnemar's Test P-Value : < 2.2e-16

Sensitivity : 0.9160

Specificity : 0.9172

Pos Pred Value : 0.5809

Neg Pred Value : 0.9887

Prevalence : 0.1113

Detection Rate : 0.1020

Detection Prevalence : 0.1755

Balanced Accuracy : 0.9166

**3. Achieving 95% sensitivity**

Confusion Matrix and Statistics

| prediction | Alive | Dead |
| --- | --- | --- |
| Alive | 1165 | 1093 |
| Dead | 73 | 8791 |

Accuracy : 0.8952

95% CI : (0.8893, 0.9008)

No Information Rate : 0.8887

P-Value [Acc > NIR] : 0.01499

Kappa : 0.6105

Mcnemar's Test P-Value : < 2e-16

Sensitivity : 0.9410

Specificity : 0.8894

Pos Pred Value : 0.5159

Neg Pred Value : 0.9918

Prevalence : 0.1113

Detection Rate : 0.1047

Detection Prevalence : 0.2030

Balanced Accuracy : 0.9152

## Supplemental discussion S2. Description of software

We used R and RStudio to perform all analysis.

The code can be retrieved by submitting a request to fredrik.hessulf@gu.se

**The session info follows:**

R version 4.1.3 (2022-03-10)

Platform: x86_64-apple-darwin17.0 (64-bit)

Running under: macOS Monterey 12.6

attached base packages:

[1] parallel stats graphics grDevices utils datasets methods base

other attached packages:

[1] table1_1.4.2 labelled_2.9.0 missRanger_2.1.3 gt_0.5.0 tableone_0.13.0

[6] randomForest_4.7-1 doParallel_1.0.17 iterators_1.0.14 foreach_1.5.2 mlbench_2.1-3

[11] caret_6.0-91 lattice_0.20-45 tictoc_1.0.1 lubridate_1.8.0 forcats_0.5.1

[16] stringr_1.4.1 dplyr_1.0.10 purrr_0.3.4 tidyr_1.2.0 tibble_3.1.8

[21] ggplot2_3.3.5 tidyverse_1.3.2 reshape2_1.4.4 readxl_1.3.1 readr_2.1.2

[26] pROC_1.18.0

loaded via a namespace (and not attached):

[1] mlr_2.19.0 backports_1.4.1 fastmatch_1.1-3 plyr_1.8.7

[5] splines_4.1.3 listenv_0.8.0 digest_0.6.29 htmltools_0.5.2

[9] fansi_1.0.3 magrittr_2.0.3 checkmate_2.0.0 BBmisc_1.12

[13] googlesheets4_1.0.0 tzdb_0.2.0 recipes_0.2.0 globals_0.14.0

[17] modelr_0.1.8 gower_1.0.0 hardhat_0.2.0 beepr_1.3

[21] colorspace_2.0-3 rvest_1.0.2 mitools_2.4 haven_2.4.3

[25] xfun_0.30 crayon_1.5.1 jsonlite_1.8.0 survival_3.3-1

[29] zoo_1.8-9 glue_1.6.2 survminer_0.4.9 gtable_0.3.0

[33] gargle_1.2.0 ipred_0.9-12 car_3.0-12 future.apply_1.8.1

[37] abind_1.4-5 scales_1.1.1 DBI_1.1.3 rstatix_0.7.0

[41] Rcpp_1.0.9 xtable_1.8-4 proxy_0.4-26 km.ci_0.5-2

[45] Formula_1.2-4 stats4_4.1.3 lava_1.6.10 survey_4.1-1

[49] prodlim_2019.11.13 httr_1.4.2 FNN_1.1.3 ellipsis_0.3.2

[53] pkgconfig_2.0.3 ParamHelpers_1.14 nnet_7.3-17 sass_0.4.1

[57] dbplyr_2.1.1 utf8_1.2.2 tidyselect_1.1.2 rlang_1.0.6

[61] munsell_0.5.0 cellranger_1.1.0 tools_4.1.3 xgboost_1.5.2.1

[65] cli_3.4.1 generics_0.1.3 audio_0.1-10 ranger_0.13.1

[69] broom_1.0.1 evaluate_0.15 fastmap_1.1.0 yaml_2.3.5

[73] ModelMetrics_1.2.2.2 knitr_1.38 fs_1.5.2 survMisc_0.5.5

[77] visdat_0.5.3 future_1.24.0 nlme_3.1-155 xml2_1.3.3

[81] compiler_4.1.3 rstudioapi_0.13 e1071_1.7-9 ggsignif_0.6.3

[85] reprex_2.0.1 stringi_1.7.6 Matrix_1.4-1 KMsurv_0.1-5

[89] vctrs_0.4.1 pillar_1.8.1 lifecycle_1.0.2 data.table_1.14.2

[93] R6_2.5.1 gridExtra_2.3 parallelly_1.30.0 codetools_0.2-18

[97] MASS_7.3-56 assertthat_0.2.1 withr_2.5.0 naniar_0.6.1

[101] hms_1.1.1 grid_4.1.3 rpart_4.1.16 timeDate_3043.102

[105] class_7.3-20 rmarkdown_2.13 carData_3.0-5 googledrive_2.0.0

[109] parallelMap_1.5.1 ggpubr_0.4.0 MLeval_0.3

## Supplemental table S7. Baseline data using imputed data set.

|  | Non-imputed data | Imputed data |
| --- | --- | --- |
| Female Sex, n (%) | 18881 (34.0) | 18916 (34.0) |
| Age yr (mean) ((SD)) | 68.88 (17.92) | 68.88 (17.92) |
| **Cause of OHCA** **– n (%)** |  |  |
| Cardiac | 30816 (62.5) | 36002 (64.7) |
| Overdose | 1427 (2.9) | 1571 (2.8) |
| Accident | 1139 (2.3) | 1297 (2.3) |
| Pulmonary disease | 2765 (5.6) | 2948 (5.3) |
| Asphyxia | 1286 (2.6) | 1486 (2.7) |
| Suicide* | 1121 (2.3) | 1126 (2.0) |
| Drowning | 465 (0.9) | 474 (0.9) |
| Sudden infant death | 170 (0.3) | 177 (0.3) |
| Other | 10155 (20.6) | 10534 (18.9) |
| Year (mean (SD)) | 2015.16 (3.08) | 2015.16 (3.08) |
| Time of day. (%) |  |  |
| 0 to 6 am | 7630 (16.5) | 8945 (16.1) |
| 1 to 6 pm | 14015 (30.3) | 16973 (30.5) |
| 7 to 11 pm | 8847 (19.1) | 10343 (18.6) |
| 7 to 12 am | 15809 (34.1) | 19354 (34.8) |
| **Location of cardiac arrest – n (%)** |  |  |
| Home | 39661 (71.6) | 39835 (71.6) |
| Public location | 8989 (16.2) | 9052 (16.3) |
| Other locations | 6728 (12.1) | 6728 (12.1) |
| Sports = No (%) | 616 (2.8) | 54980 (98.9) |
| Collapse to EMS dispatch | 2.00 [1.00, 5.00] | 2.00 [1.00, 5.00] |
| Collapse to CPR | 3.00 [0.00, 10.00] | 3.00 [0.00, 10.00] |
| Collapse to first defibrillation | 15.00 [8.00, 24.00] | 15.00 [8.00, 24.00] |
| Time from collapse to EMS arrival | 13.00 [8.00, 20.00] | 13.00 [8.00, 20.00] |
| Time from EMS dispatch to EMS arrival | 10.00 [7.00, 16.00] | 10.00 [7.00, 16.00] |
| Time to ROSC | 15.00 [9.00, 23.00] | 15.00 [9.00, 23.00] |
| Witnessed CA | 34923 (64.7) | 35570 (64.0) |
| CA witnessed by EMS | 3916 (21.3) | 7098 (12.8) |
| Telephone CPR | 9565 (62.7) | 44246 (79.6) |
| Training CPR provider |  |  |
| Layperson without CPR training | 5389 (46.9) | 21627 (38.9) |
| Layperson with CPR training | 4215 (36.7) | 20714 (37.2) |
| Health care worker | 1891 (16.5) | 13274 (23.9) |
| EMS first on scene (%) | 22255 (74.5) | 41264 (74.2) |
| Fire department first on scene (%) | 8940 (31.1) | 13418 (24.1) |
| Police first on scene (%) | 1373 (4.9) | 1393 (2.5) |
| **Initial Rhythm** |  |  |
| VF/pVT, | 11374 (23.2) | 13033 (23.4) |
| PEA, | 8421 (17.2) | 9144 (16.4) |
| Asystole | 29248 (59.6) | 33438 (60.1) |
| Patient conscious at EMS arrival at scene | 5728 (10.6) | 6097 (11.0) |
| Patient breathing at EMS arrival at scene |  |  |
| No breathing | 42007 (77.7) | 43312 (77.9) |
| Agonal breathing | 5791 (10.7) | 5793 (10.4) |
| Normal breathing, | 6212 (11.5) | 6488 (11.7) |
| Unknown, | 22 (0.0) | 22 (0.0) |
| Pulse at EMS arrival at scene | 7384 (14.0) | 7659 (13.8) |
| Return of spontaneaus circulation at hospital arrival | 14190 (44.7) | 14802 (26.6) |
| Conscious at hospital arrival | 3418 (11.0) | 15775 (28.4) |
| Bystander treatment before EMS arrival - Yes | 29491 (55.1) | 30520 (54.9) |
| CPR before EMS arrival -Yes | 13488 (57.3) | 30885 (55.5) |
| Bystander def. with public AED – Yes | 1863 (6.6) | 1863 (6.6) |
| Mechanical CPR | 21486 (40.4) | 21566 (38.8) |
| Ventilation | 51142 (92.6) | 51448 (92.5) |
| Endotracheal intubation | 15437 (28.3) | 15504 (27.9) |
| Laryngeal Mask | 19960 (61.0) | 35041 (63.0) |
| Defibrillation | 17812 (33.3) | 17812 (33.3) |
| Number of defibrillations, n (mean (SD)) | 3.48 (3.16) | 3.48 (3.16) |
| Adrenalin | 43290 (78.8) | 43715 (78.6) |
| Amiodarone | 6365 (11.8) | 6370 (11.5) |

We note the discrepancy in rates of ROSC and consciousness when comparing imputed and non-imputed data. Therefore, we reran the analysis with non-imputed data. The ROC-value was 0.9526 with non-imputed data ie very similar to imputed data.
